# Integrating ATAC‐Seq and Pan‐Genomics Identifies Stress‐Memory *AP2/ERF* Hubs in Foxtail Millet

**DOI:** 10.1002/fsn3.71109

**Published:** 2025-11-29

**Authors:** Tingting Li, Wenhui Liao, Hao Wang, Zi Wang, Jiayi Li, Xue Zhou, Yongmao Cai, Jiacheng Zhang, Fan Feng, Youcai Wang, Wenjiang Wang, Junpeng Hu, Yulin Sun

**Affiliations:** ^1^ College of Bioengineering Shaanxi A&F Technology University Yangling Shaanxi China; ^2^ State Key Laboratory of Crop Stress Biology in Arid Areas, College of Agronomy Northwest A&F University Yangling Shaanxi China; ^3^ Yan'an Academy of Agricultural Sciences Yan'an Shaanxi China; ^4^ Department of Botany University of British Columbia Vancouver British Columbia Canada

**Keywords:** *AP2/ERF*, drought memory, *foxtail millet*, pan‐genomics

## Abstract

Foxtail millet ( *Setaria italica* ), a key cereal crop, has developed robust mechanisms to adapt to drought conditions. Investigating genes associated with drought memory is crucial for improving the plant's resilience against recurring drought events. Our study leveraged ATAC‐seq and RNA‐seq to reveal significant changes in chromatin accessibility and gene expression in response to secondary drought stress. The *AP2/ERF* transcription factor motifs were the most enriched within different chromatin regions in both leaf and root tissues. Transcriptome analysis identified 80 significantly upregulated *AP2/ERF* genes in foxtail millet. Pan‐genome analysis of 111 accessions revealed 16,778 *AP2/ERF* genes, categorized into 17 groups, highlighting gene number variations across populations. Codon usage analysis showed biased preferences across groups and populations. Natural selection studies indicated that most *AP2/ERF* genes are under strong purifying selection (Ka/Ks < 1), while dispensable genes exhibit greater evolutionary flexibility. Collinearity analysis has validated the conservation of 107 *AP2/ERF* genes across four Poaceae species, underscoring their ancient and critical functions in drought response. Of particular interest, 57 of these conserved genes were significantly upregulated in the leaves or roots under secondary drought stress, with *SiERF‐51* showing the highest expression levels in both tissues, a finding verified by qRT‐PCR analysis. This study not only demonstrated the pivotal role of the *AP2/ERF* superfamily in drought memory but also provides a wealth of genetic resources for further investigations into the molecular mechanisms of drought adaptation in foxtail millet.

## Graphical abstract

The *AP2/ERF* super gene family was identified through ATAC sequencing as playing a crucial role in the drought memory response. Through pan‐genomic analysis of 111 *foxtail millet* accessions, we identified a total of 16,778 *AP2/ERF* genes and constructed an orthologous gene group containing 125 core and softcore genes. These genes exhibited significant conservation across different foxtail millet populations, indicating their important functions in the species' evolution. Collinearity analysis further validated the conservation of 107 *SiAP2/ERF* genes across four *Poaceae* species, highlighting their ancient and critical roles in drought response. Under secondary drought stress, 57 *SiAP2/ERF* genes were significantly upregulated, with *SiERF‐51* showing the highest expression levels in both roots and leaves, a finding confirmed by qRT‐PCR analysis. This study not only revealed the pivotal role of the AP2/ERF superfamily in drought memory but also provided a wealth of genetic resources for further investigations into the molecular mechanisms of drought adaptation in foxtail millet, offering a theoretical basis and potential gene targets for breeding more drought‐resistant foxtail millet varieties.

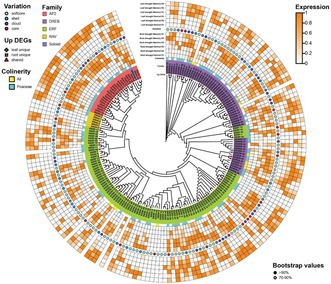

## 1 Introduction

During the growing season, plants typically experience a series of milder drought events interspersed with recovery phases where water is replenished (Ruehr et al. 2019). Against the backdrop of climate change, the frequency and intensity of such recurring droughts have increased (Stott 2016). Repeated drought events throughout the growing season may impact plants differently compared to singular stress events (Fleta‐Soriano and Munné‐Bosch 2016). Consequently, it is believed that plants adapt to repeated stresses by increasing their tolerance to ensure the maintenance of growth and reproduction (Schulze et al. 2021). The Assay for Transposase‐Accessible Chromatin using sequencing (ATAC‐seq) technology has been successfully applied in the study of various plants to explore key transcription factors related to stress tolerance. For instance, in apple research, ATAC‐seq combined with RNA‐seq revealed that *MdRAD5B* affects the expression and chromatin accessibility of 466 drought‐responsive genes under drought stress (Song et al. 2024). Furthermore, *ERF5* in maize was identified as emerging pivotal in the cold response regulatory network by using ATAC‐seq data (Han et al. 2025). This highlights the crucial role of ATAC in detecting key transcription factors under stress conditions.

Plants have the ability to remember the chromatin state under drought conditions to cope with repeated stresses (He et al. 2024; Jacques et al. 2021; Nguyen et al. 2022). This “memory” capability is reflected in plants that have been pre‐exposed to drought, showing enhanced tolerance and improved photosynthetic efficiency under low water conditions. During the initial stress, plants establish stress imprints through physiological and molecular mechanisms, such as the accumulation of osmotic regulatory substances or the synthesis of protective proteins (Avramova 2019). During the recovery period, these imprints enable plants to shift into a tolerant state, thus responding more rapidly and robustly to subsequent stresses (He et al. 2024; Jacques et al. 2021; Nguyen et al. 2022). Research indicates that after multiple exposures to drought, *Arabidopsis thaliana* and maize exhibit transcriptional stress memory, including *AP2/ERF* and *NAC* families (Ding et al. 2014; Jacques et al. 2021). The transcription factors may play a central role in bridging biotic and abiotic stress responses for plant resilience.

The APETALA2/Ethylene Responsive Factor (*AP2/ERF*) superfamily acts as key molecular switches, modulating complex transcriptional cascades in response to abiotic stresses (Wang et al. 2024; Xie et al. 2019). AP2/ERF contains one or two AP2 DNA‐binding domains that bind to specific promoter motifs, such as the dehydration‐responsive element (DRE/CRT) or the GCC box (Agarwal et al. 2017; Ghorbani et al. 2020; Zarei et al. 2011). The superfamily is classified into *AP2*, *DREB*, *ERF*, *RAV*, and *Soloist* families (Dossa et al. 2016; Feng et al. 2020). *DREBs* primarily participate in responses to dehydration (Narusaka et al. 2003), cold (Lata and Prasad 2011), and salinity (Narusaka et al. 2003) via DRE/CRT binding, with roles confirmed for *PgDREB2A* and *GuDREB35* in enhancing abiotic stress tolerance (Meena et al. 2022; Yang et al. 2025). ERFs bind to the GCC box and are linked to ethylene signaling (Chen et al. 2020; Fujimoto et al. 2000), biotic stress (Cheng et al. 2013; Nie and Wang 2023), and abiotic stress responses (Klay et al. 2018). For instance, *GmAP2/ERF144* from soybean enhances drought tolerance when overexpressed in Arabidopsis (Wang et al. 2022), while *FaTINY2* from strawberry improves both cold and salt tolerance (Li et al. 2025). From model organisms to a variety of crops, *AP2/ERF* genes have been identified across diverse species, including Medicago (Haddoudi et al. 2025), Prunus (Zhang, Zeng, et al. 2025), pomegranate (Wan et al. 2022), lettuce (Park et al. 2023), wheat (Riaz et al. 2021), Rhododendron (Guo et al. 2023), peanut (Cui et al. 2021), soybean (Wang et al. 2022), citrus (Zhang, Zi, et al. 2025), strawberry (Su et al. 2022), rose (Li et al. 2020), kiwifruit (Jiang et al. 2022), tea plant (Liu et al. 2023), and *Brachypodium* (Huang et al. 2024). Demonstrated roles of specific *AP2/ERF* factors in improving stress tolerance through genetic manipulation highlight their potential for crop improvement (Huang et al. 2024; Ma et al. 2024; Meena et al. 2022; Yang et al. 2025).

Foxtail millet, renowned for its drought tolerance and compact genome, stands as an exemplary model for exploring the molecular underpinnings of drought adaptation in C4 crops (He et al. 2024). Although numerous studies on drought resistance have been reported, research on drought memory in foxtail millet is still limited. Recent advancements in genomic resources, such as the updated “Yugu1” reference telomere to telomere (T2T) genome (He et al. 2024) and pan‐genome construction (He et al. 2023), have provided a rich source of data. By harnessing these resources, we can investigate the variability of key transcription factors at the pan‐genome level.

In this study, we first utilized ATAC‐seq and RNA‐seq to pinpoint the key transcription factors of the *AP2/ERF* family involved in drought memory and then constructed the pan‐genome profile of the *AP2/ERF* family using three populations of 111 accessions. Finally, one core gene, *SiERF‐51*, showing the most prominently upregulated gene during the second drought stress in both leaf and root tissues, was identified and verified by qRT‐PCR. The findings are expected to provide novel insights into the regulatory architecture underlying second drought tolerance in foxtail millet and identify promising candidates for future functional characterization and potential application in crop improvement strategies aimed at ensuring food security in water‐limited environments.

## 2 Material and Methods

### 2.1 Plant Materials and Drought Treatments

The study employed Jigu 27, a drought‐tolerant foxtail millet cultivar developed by the Shandong Academy of Agricultural Sciences. Seeds were surface‐sterilized, germinated on moist filter paper, and transferred to half‐strength Hoagland's solution. Seedlings were grown under controlled conditions (28°C day/20°C night, 16‐h light/8‐h dark photoperiod). Three experimental groups were established (Figure S1): (I) direct drought: exposed to 19.2% PEG‐6000; (II) drought memory: subjected to three 24‐h cycles of 10% PEG pretreatment, each followed by 24‐h recovery, prior to the final 19.2% PEG treatment. At 6 h post‐final drought treatment, shoots and roots were collected separately and flash‐frozen in liquid nitrogen for parallel transcriptomic and ATAC‐seq analyses.

### 2.2 Transcriptome and ATAC Sequencing

Total RNA was extracted from 12 samples using TRIzol Reagent (Invitrogen, USA) and treated with DNase I to remove genomic DNA. RNA integrity was verified using an Agilent Bioanalyzer 2100 system (RIN ≥ 8.0), and quantity was measured by Qubit fluorometry (Thermo Fisher Scientific). For library construction, 1 μg of total RNA per sample was processed using the NEBNext Ultra RNA Library Prep Kit for Illumina (NEB, USA). Poly (A) selection was used to enrich mRNA, followed by fragmentation to an insert size of approximately 300 bp. Adapter‐ligated cDNA was amplified with 12 PCR cycles. Final library quality was assessed using an Agilent Bioanalyzer (size distribution: 350–450 bp) and quantified by qPCR (KAPA Biosystems). Paired‐end sequencing (150 bp) was carried out by Kindstar Sequenon (Wuhan, China) using an Illumina NovaSeq 6000 system.

For the ATAC‐seq, fresh leaves and roots were collected and subjected to mechanical homogenization in cold NP‐40 lysis buffer (10 mM Tris–HCl, pH 7.5; 10 mM NaCl; 3 mM MgCl_2_; 0.1% NP‐40) to isolate nuclei. The isolated nuclei were then treated with Tn5 transposase (Illumina Tagment DNA Enzyme) at 37°C for 30 min to induce tagmentation. The transposed DNA was purified using AMPure XP beads at a 1.8× ratio, followed by ligation to Illumina adapters and PCR amplification (12 cycles; NEBNext High‐Fidelity Master Mix). Size selection of the libraries was performed to obtain fragments ranging from 150 to 700 bp. These libraries were quantified using a Qubit 4.0 fluorometer and quality checked using an Agilent 2100 Bioanalyzer. The equimolar pooled libraries were then clustered on a cBot System using a TruSeq PE Cluster Kit and sequenced on an Illumina HiSeq X Ten platform (150 bp paired‐end) by Kindstar Sequenon (Wuhan, China).

### 2.3 RNA‐Seq and ATAC‐Seq Data Analysis

Raw reads from RNA and ATAC sequencing are processed to remove adapter sequences and filter out low‐quality reads by fastp v0.23.2 (Chen et al. 2018). Subsequently, clean reads from RNA‐seq were aligned to the foxtail millet Yugu1 reference genome using HISAT2 v2.2.1 (Kim et al. 2015). The expression level quantified using FPKM (Fragments Per Kilobase of transcript per Million mapped reads) was determined by StringTie v2.2 (Pertea et al. 2015). The Differentially expressed genes were identified using DESeq2 (Love et al. 2014), with padj < 0.05 and |log_2_FoldChange| > 1 used to determine the significance of gene expression difference.

The ATAC‐seq clean reads were aligned to the Yugu1 reference genome by bwa v0.7.17 (Li and Durbin 2009). Unique mapped reads were identified, and redundant alignment results caused by PCR amplification were removed by SAMtools v.1.9 (Li et al. 2009). Peak calling was then conducted using MACS v2.1.1 (Zhang et al. 2008). Genomic locations of these peaks are annotated by ChIPseeker v1.1.16 (Yu, Wang, and He 2015). The DiffBind v2.14 (Ross‐Innes et al. 2012) package was utilized for differential peak analysis with |log_2_FoldChange| > = 2 and FDR < = 0.01.

### 2.4 Pan‐Genome‐Wide Identification of AP2/ERF Superfamily in Foxtail Millet

The genome sequences of 111 foxtail millet accessions were downloaded from Setaria DB (He et al. 2024) (http://111.203.21.71:8000/index.html). A hidden Markov model (HMM) profile AP2 hidden Markov model (PF00847) was conducted to identify *AP2/ERF* genes using HMMER v3.0.16 (Finn et al. 2011). All retrieved protein sequences containing the AP2 domain were verified by a batch search of the NCBI Conserved Domain Database (Marchler‐Bauer et al. 2015). To characterize the *SiAP2/ERF* superfamily at the pan‐genome level, we employed a method that iteratively scans for the presence or absence of *AP2/ERF* genes on the reference *AP2/ERF* gene set. Specifically, the nucleotide coding sequences of *SiAP2/ERF* genes from the Yugu1 T2T genome were used as the initial reference sequences. We then compared candidate *AP2/ERF* gene sequences identified in other varieties to these references using BLASTn (Altschul et al. 1990), with a cutoff of 90% identity and 90% alignment coverage. Sequences meeting these criteria were grouped with their corresponding reference sequences. For sequences that did not align, we selected the most abundant sequences from one variety as a new reference for the next round of comparisons. This iterative process continued until no new sequences were added, and 236 *SiAP2/ERF* genes were selected. We obtained the *AtAP2/ERF* sequences from the Arabidopsis Information Resource (TAIR, http://www.arabidopsis.org/) and integrated these with the 236 *SiAP2/ERF* genes to construct phylogenetic ML trees using iqtree2 (Minh et al. 2020) and visualized by Evolview (Subramanian et al. 2019). This enabled us to classify the *SiAP2/ERF* superfamily into five distinct families (*AP2*, *DREB*, *ERF*, *RAV*, and *Soloist*) based on *AtAP2/ERF* classification. For the *AP2* gene family, the presence of two AP2 domains was mandatory. For the *RAV* family, one AP2 domain along with one B3 domain (PF02362) was required. Genes in the remaining families needed to possess one AP2 domain. After applying this filtering process, we ultimately identified a total of 225 nonredundant *SiAP2/ERF* genes, which correspond to 225 orthologous gene groups (OGGs) encompassing 16,778 *SiAP2/ERF* genes across 111 accessions of foxtail millet.

### 2.5 Estimation of Codon Bias of SiAP2/ERF

Utilizing the online platform CAIcal (Puigbò et al. 2008), the codon usage of 17 SiAP2/ERF group genes in three populations was assessed. The assessment parameters included the Codon Adaptation Index (CAI), the total GC content of the coding genes, the Effective Number of Codons (ENC), the GC content at the first codon position (GC1), the second codon position (GC2), and the third codon position (GC3). The CAI indicates how closely the frequency of synonymous codon usage in the coding region approaches the frequency of optimal codon usage, with values ranging from 0 to 1, where higher values suggest usage closer to the optimal pattern (Sharp and Li 1987). We use all the genes with FPKM > 10 for the reference for CAI calculation. The ENC measures the preference for codon usage within a gene, reflecting the degree of nonrandom selection among synonymous codons, with values ranging between 20 and 61, and lower values indicate a stronger preference for codon usage (Fuglsang 2004).

### 2.6 Core and Dispensable Analysis

Based on their frequency of the 111 *Setaria* accessions on the 225 OGGs, they were categorized into four distinct groups based on their frequency: core (these present in all 111 accessions), soft core (these present in > 90% of accessions but not all; 100–110 accessions), shell (these present in more than 10% but less than 90%; 12–99 accessions), cloud (these present in more than 1 but less than 90%; 2–11 accessions). To calculate pair‐wise gene Ka, Ks, and Ka/Ks values among each OGGs, the ParaAT tool (v 2.0) (Zhang et al. 2012) was used to construct the pair‐wise sequence alignment and then calculated using the KaKs_calculator (Zhang 2022). Ka/Ks values (*p* value < 0.01) were compared among the core, soft core, and other dispensable genes using the Wilcoxon test.

### 2.7 Collinear Analysis

We utilized BLASTP to identify homologous genes with a set *E*‐value threshold of 1e‐3 and limiting the search to a maximum of 10 target sequences. For the identification of collinear genes, MCScanX (Wang et al. 2012) was employed with default settings. The Ka and Ks values were calculated using the KaKs_calculator (Zhang 2022).

### 2.8 Quantitative Real‐Time PCR Analysis

To confirm the findings from the RNA‐seq analysis, eight differentially expressed genes with significant changes were selected for validation via quantitative real‐time PCR (qRT‐PCR). Primers for the candidate DEGs and the housekeeping gene *cullin* (*Seita.3G037700*) were designed using Primer 5.0 software, as shown in Table S1. Total RNA was separately extracted from leaves and roots using the MiniBEST Plant RNA Extraction Kit (TaKaRa, Beijing, China) and assessed for quality using a NanoDrop 2000 spectrophotometer (Thermo Fisher Scientific) and agarose gel electrophoresis. First‐strand cDNA was synthesized from 1 μg of RNA using the PrimeScript RT Reagent Kit (TaKaRa) with oligo(dT) and random hexamer primers. qRT‐PCR was performed on a Bio‐Rad CFX96 Real‐Time PCR Detection System using TB Green Premix Ex Taq II (TaKaRa). The 20 μL reaction mixture contained 0.2 μM of each gene‐specific primer and 2 μL of 5× diluted cDNA template. Three biological replicates were analyzed per condition, with each sample run in technical triplicates. Relative gene expression was calculated using the $2^{-\Delta \Delta C_{T}}$ method (Livak and Schmittgen 2001).

## 3 Results

### 3.1 Chromatin Accessibility Profiles of Foxtail Millet Involved in Drought Recovery and Memory

**FIGURE 1:**
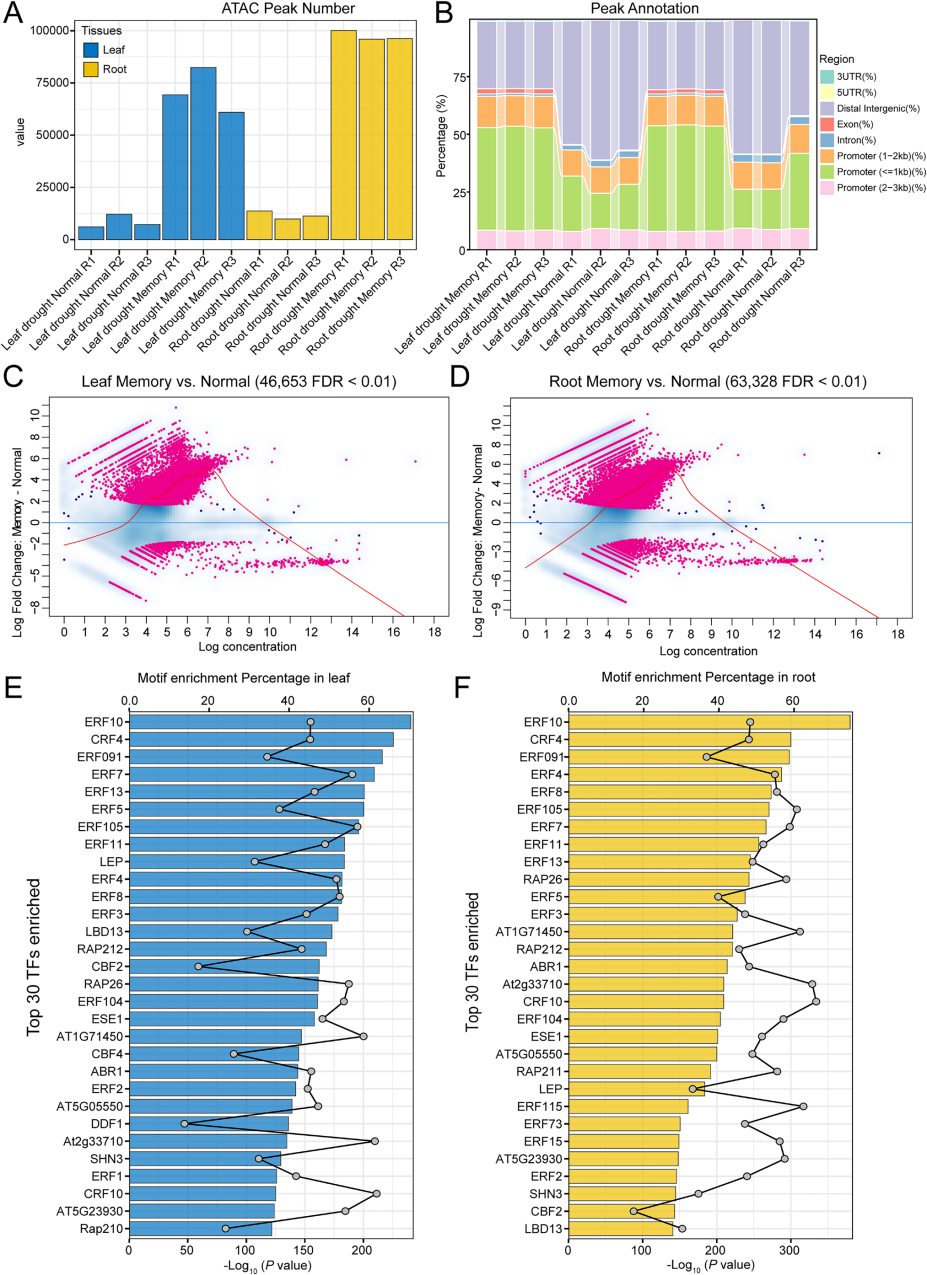
Chromatin accessibility landscape in leaves and roots of foxtail millet under second drought stress. (A) Number of ATAC peaks called in leaves and root tissues. (B) Genomic annotations of peaks. (C, D) MA plots. The *X*‐axis represents the average ATAC signal abundance in the region, while the *Y*‐axis shows the log_2_ difference in ATAC signal between the two conditions. Black dots indicate non‐significant regions, and red dots highlight significant differentially accessible regions. Blue lines represent loess fits to each distribution, with 95% confidence intervals shaded in gray. (E, F) Top 30 motifs enriched in DARs. Bar charts display the enrichment *p*‐values of the motifs, corresponding to the lower axis scale. Line charts show the proportion of motifs enriched in target sequences, corresponding to the upper axis scale.

In this study, we conducted ATAC‐seq experiments on leaf and root samples under drought recovery (referred to as drought normal) and secondary drought (referred to as drought memory) conditions to investigate the drought memory mechanism of foxtail millet (Figure S1). Through ATAC‐seq sequencing, we obtained a total of 35.55 million (M) to 42.07 M reads (Table S2). We called a large number of peaks in both the drought normal and drought memory groups. Specifically, the normal leaf group had an average of 8502 peaks, while the drought memory group had 70,888 peaks (Figure 1A). In the roots, the normal group had 11,613 peaks on average, compared to 97,441 in the drought memory group (Figure 1A). Peak annotation analysis showed that these were widely distributed across different genomic regions, including promoters, gene bodies, and intergenic regions. Notably, peak distribution exhibited significant differences after secondary drought stress (Figure 1B). For instance, in the recovered drought normal leaves, the proportion of peaks located in promoter regions (< 1 kb) was less than half that of the drought memory group leaves (19.66% in the drought normal group vs. 44.71% in the drought memory group). Similarly, in the roots, the proportion of promoter peaks decreased to 22.35% in the normal group compared to 45.79% in the memory group. These findings indicate that drought stress induces significant alterations in chromatin accessibility patterns across different tissues of *foxtail millet*.

In terms of differential peak analysis, we identified 46,653 and 63,328 significantly DARs (differentially accessible regions) in leaves and roots, respectively. The majority of these peaks were upregulated in the drought memory group for both tissues (Figure 1C,D), consistent with the significant increase in peak numbers in this group (Figure 1A). To uncover transcriptional regulators involved in the drought stress response, we identified DNA motifs enriched in the DARs (*E*‐value < 0.05). We detected 190 motifs in leaves (Table S3) and 181 in roots (Table S4). Notably, the AP2/ERF transcription factor family was significantly overrepresented among the top 30 most enriched motifs in both tissues (Figure 1E,F), with 90% of the transcription factors identified belonging to this family, which suggested they may play important potential roles in drought memory response. In addition, *LBD13* and *Trihelix* were detected in leaf or root tissues.

### 3.2 Transcriptome Profiles of Foxtail Millet Involved in Drought Recovery and Memory

**FIGURE 2:**
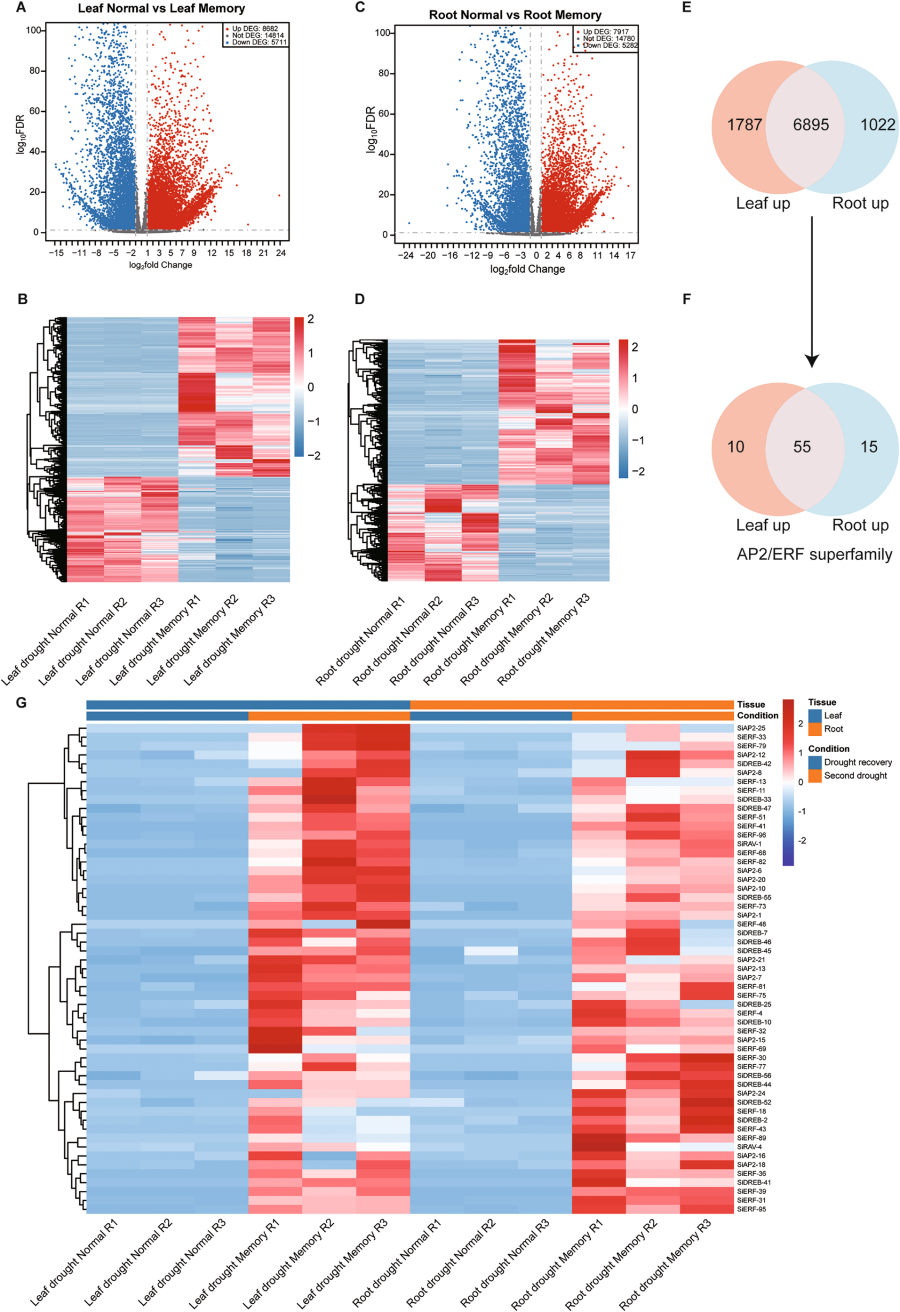
Transcriptional profiling of *foxtail millet* leaves and roots under second drought stress. Volcano and heatmap plots display differentially expressed genes between drought‐normal and drought‐memory conditions in leaves (A, B) and roots (C, D). (E) A Venn diagram illustrates the number and overlap of upregulated DEGs between leaves and roots. (F) A Venn diagram shows the intersection of upregulated genes from the AP2/ERF family in roots and leaves. (G) Expression heatmap for shared upregulated *AP2/ERF* genes from Yugu1 cultivar.

We performed transcriptomic sequencing on leaves and roots during drought recovery and secondary stress, generating a total of 281 M reads across 12 samples (Table S2). Differential expression analysis revealed that in leaves, 8682 genes were significantly upregulated and 5711 genes were downregulated (Figure 2A,B, and Table S5). In roots, 7917 genes were upregulated and 5282 genes were downregulated (Figure 2C,D, and Table S6). Further analysis identified 6895 genes that were upregulated in both leaves and roots, representing 71.05% of all upregulated differentially expressed genes (DEGs) (Figure 2E). This observation highlights the prevalence of a shared gene expression pattern between these tissues, which likely underpins the coordinated response strategy of plants to drought stress. GO enrichment of the shared upregulated genes shows these genes enriched in pathways such as protein phosphorylation, response to oxidative stress, and hydrogen peroxide catabolic process (Figure S2A). The KEGG analysis results indicate that plant–pathogen interaction and plant hormone signal transduction were significantly enriched (Figure S2B).

The ATAC‐seq analysis above indicates that the AP2/ERF superfamily plays a pivotal role in drought stress memory. This finding has prompted us to focus our analysis on this family. We first identified 187 members of the AP2/ERF superfamily in the T2T genome of Yugu1 (Table S7), and 80 were upregulated DEGs (Figure 2F). Notably, among the shared upregulated DEGs, 55 members of the AP2/ERF transcription factor family were identified (Figure 2F,G). This finding further underscores the pivotal role of the AP2/ERF superfamily in orchestrating the expression of drought‐responsive genes.

### 3.3 Pangenome‐Wide Identification of the AP2/ERF Superfamily in *Foxtail Millet*

**FIGURE 3:**
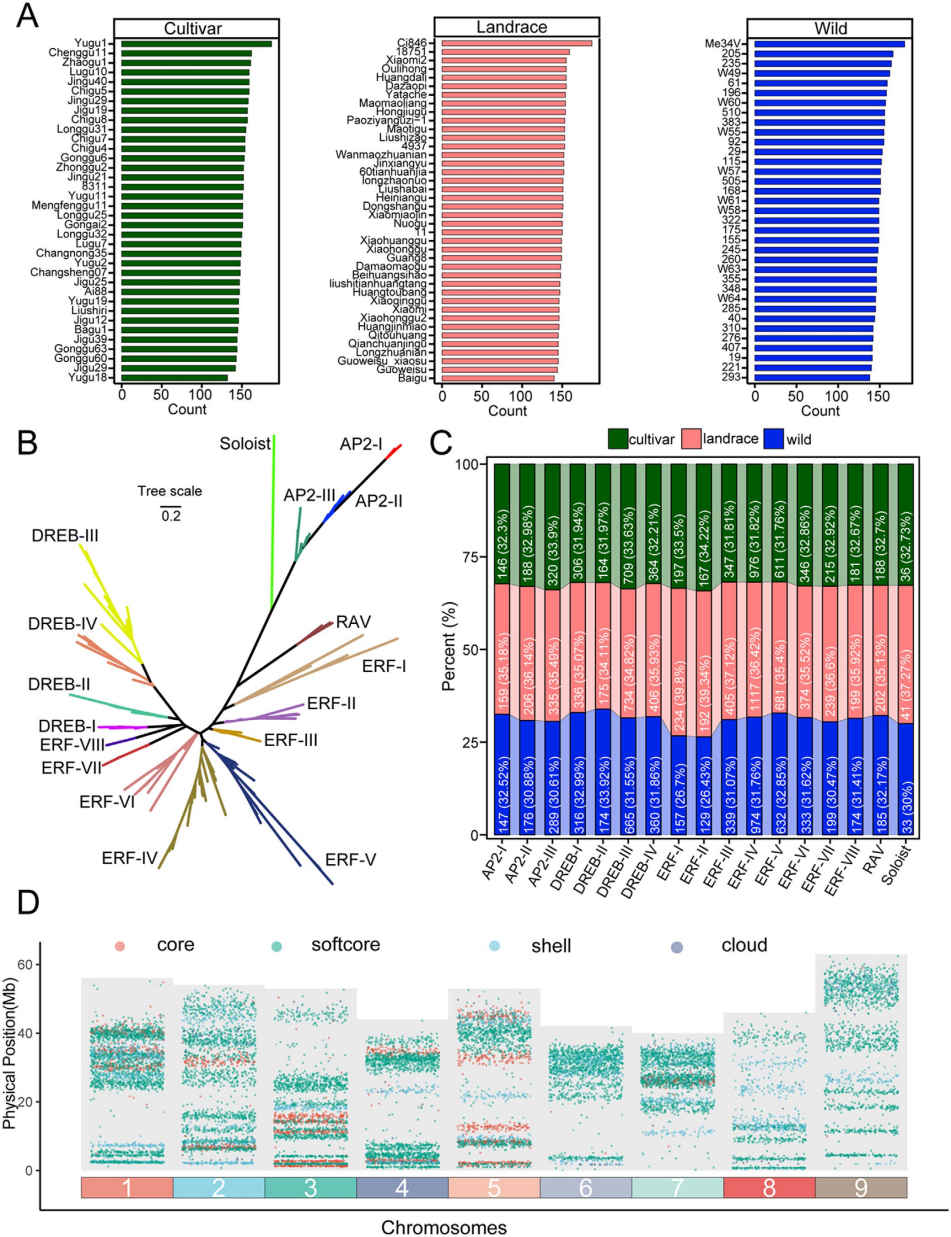
Identification and distribution analysis of *SiAP2/ERF* genes in 111 accessions. (A) Number of SiAP2/ERF members in each accession. (B) Pangenome‐based phylogenetic classification of 225 SiAP2/ERF gene families. (C) Proportion of gene members from cultivars, landraces, and wild varieties in each of the 13 groups. (D) Chromosomal locations of all *SiAP2/ERF* members on the chromosome genome.

To conduct an in‐depth analysis of the AP2/ERF superfamily, we examined 111 accessions (36 cultivars, 40 landraces, and 35 wild varieties) and identified a total of 16,778 *SiAP2/ERF* genes (Table S8). Within the cultivar group, Yugu1 and Yugu81 harbored the highest and lowest numbers of AP2/ERF genes, with 187 and 132 genes, respectively. In the landrace group, Ci846 had the most (187 genes), while Baigu had the fewest (140 genes). Among the wild varieties, Me34V had the highest number (180 genes), and sample 293 had the lowest (138 genes) (Figure 3A). Iterative assembly is a method for constructing a pan‐genome (Sarawad et al. 2025), which we have applied to the construction of a pan‐gene family. Specifically, we used Yugu1 as a reference genome to serve as the initial template of the *SiAP2/ERF* gene superfamily. The genomes of newly added individuals are compared with the reference gene family members, and any new *SiAP2/ERF* sequences discovered are integrated to generate an updated pan‐gene family. This process is repeated by sequentially adding other individuals, ultimately leading to the development of a comprehensive pan‐gene family that encompasses all individuals. Using the 16,778 genes identified from 111 accessions of *foxtail millet*, we constructed a pan‐gene family of *SiAP2/ERF* comprising 225 gene members. Through nucleotide sequence alignment, all *SiAP2/ERF* genes were systematically assigned to 225 OGGs (Table S9). By integrating information from the *A. thaliana AP2/ERF* gene with these 225 *SiAP2/ERF* genes, we constructed a phylogenetic ML tree and categorized the *SiAP2/ERF* superfamily into five distinct families, including *AP2*, *ERF*, *DREB*, *RAV*, and *Soloist* (Figure S3). Subsequently, we conducted a further subdivision of these 225 *SiAP2/ERF* genes into subfamilies, which resulted in a total of 17 groups (Figure 3B). Comparative analysis across different populations showed that the proportions of *ERF‐I* (cultivar: 33.5%, landrace: 39.8%, wild: 26.7%), *ERF‐II* (cultivar: 34.22%, landrace: 39.34%, wild: 26.43%), and *Soloist* (cultivar: 32.73%, landrace: 37.27%, wild: 30.00%) families increased in landrace and cultivar populations compared to the wild population (Figure 3C). This suggests that these three groups may have undergone selective expansion during domestication.

Subsequently, the chromosome locations of all *SiAP2/ERF* genes were drawn, and it was observed that core genes (defined in Section 3.5) are located on specific chromosomes, with the highest distribution on chromosomes 3 and 5, each harboring five genes (Figure 3D). For further clarification, we specifically pay attention to the T2T genome of Yugu1, which encompasses 97 *ERF* genes, 58 *DREB* genes, 25 *AP2* genes, 6 *RAV* genes, and 1 *Soloist* gene, totaling 187 genes distributed across nine chromosomes (Table S7 and Figure 3D). In terms of distribution, the single Soloist gene (*SiSoloist* −1, *Seita.1G165600*) is on chromosome 1. Chromosome 1 has the highest number of genes (31), followed by chromosome 2 with 26 genes, while chromosomes 8 and 6 have the fewest, with 12 and 14 genes, respectively.

### 3.4 Analysis of the Codon Usage Bias of *SiAP2/ERF* Genes

**FIGURE 4:**
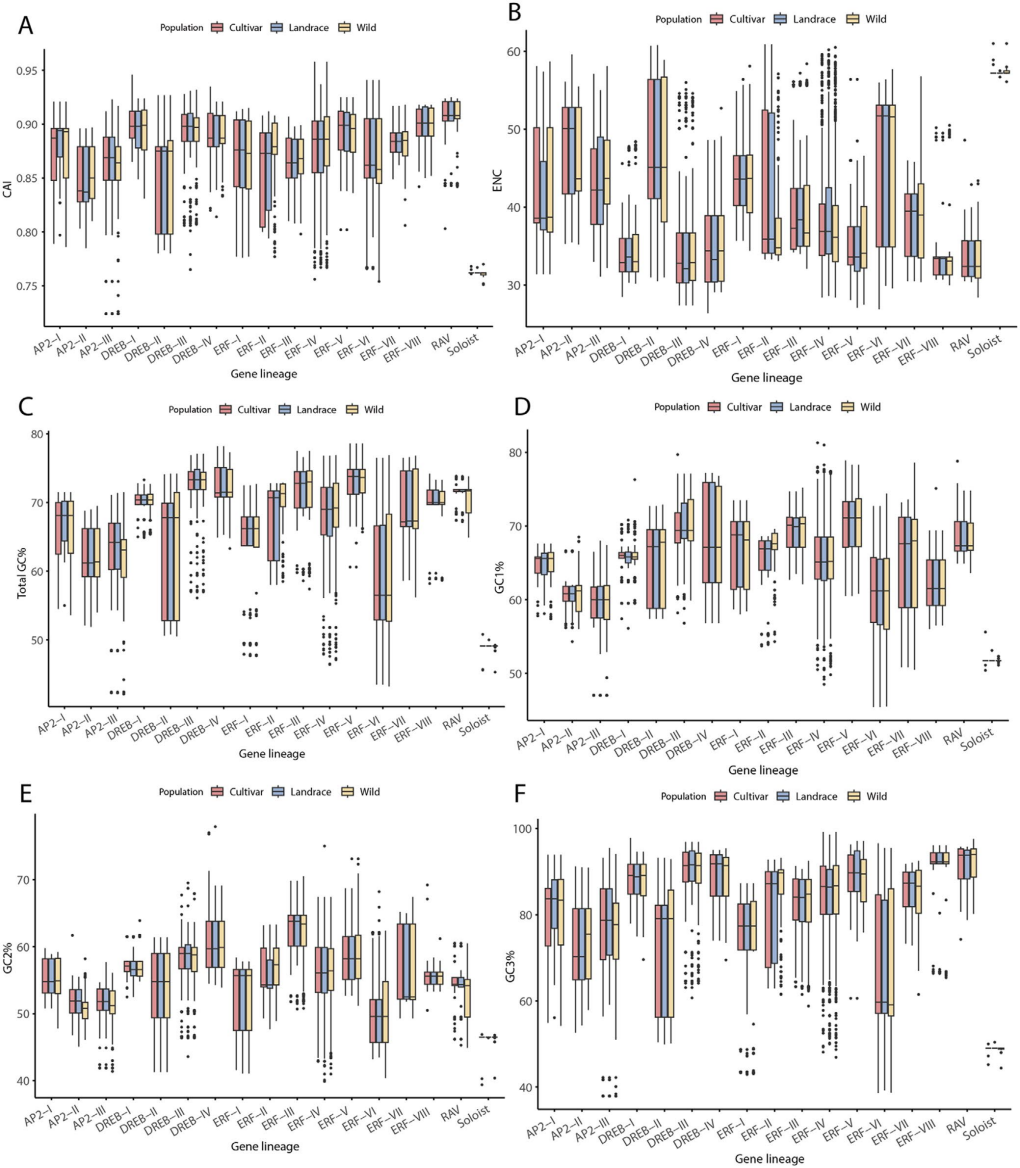
Comparative analysis of codon parameters for each group of the SiAP2/ERF superfamily across three populations. (A) CAI. (B) ENC. (C) Total GC content percentage. GC content percentage at the first codon position (D), second codon position (E), and third codon position (F).

We observed differentially preferred codons and different gene features across different groups of SiAP2/ERF and different populations (Figure 4 and Figure S4). In the Soloist family, UAG and UUG codons were predominantly employed, whereas seven other codons (CUG, AUC, CCG, GUG, CGC, GGC, and CUC) were most frequently utilized by the other SiAP2/ERF families when compared to the rest (Figure S4). We also observed that the average Relative Synonymous Codon Usage (RSCU) scores for different populations within each gene family cluster together, indicating that the codon usage preferences are similar among three populations (Figure S4). We also calculated six other codon parameters, including codon adaptation index (CAI), total GC content, effective number of codons (ENC), GC content at the first codon position (GC1), GC content at the second codon position (GC2), and GC content at the third codon position (GC3), for 16,778 coding sequences across 17 groups of AP2/ERF superfamily in three populations (Figure 4, Tables S10 and S11). We calculated the CAI for each group and found that it varied significantly among different groups, with average value range of 0.76 (Soloist) to 0.90 (RAV) (Table S10). Within the same subfamily, CAI values for most subfamilies were highly similar across different populations, except for the Soloist family, which exhibited significantly lower values in wild populations than cultivated and landrace (Figure 4A, Wilcoxon test *p* < = 0.01). The average ENC value ranged from 33.77% (DREB‐III) to 57.32% (Soloist) (Figure 4B), suggesting that there were differences among groups. Within the DREB family, DREB‐II had higher ENC values than other subfamilies, reaching above 45.29%. In the ERF family, ERF‐VI had higher ENC values than other subfamilies, with an average of around 45.07%. We observed that the ENC values of the DREB‐III and Soloist were significantly lower in cultivated and landrace varieties than in wild varieties, while the ENC values of ERF‐IV were significantly lower in wild varieties than in landrace varieties (Figure 4B and Table S11, Wilcoxon test *p* < = 0.05).

Additionally, we analyzed the GC content of all *SiAP2/ERF* genes, and most of the group genes had an average GC content exceeding 50%, except for the Soloist subfamilies (Figure 4C). We found that DREB‐II in the DREB family and ERF‐VI in the ERF family had lower GC content than other subfamilies (Figure 4C). The GC content was highly similar across different populations within the same group, except for the ERF‐II, which exhibited significantly higher values in wild varieties compared to landrace populations (Figure 4C). We also calculated the GC1, GC2, and GC3 values for each group. The average GC1 values ranged from 51.74% (Soloist) to 70.32% (ERF‐V) (Figure 4D). The average GC2 values ranged from 46.31% (Soloist) to 62.31% (ERF‐III) (Figure 4E). The average GC3 values ranged from 48.89% (Soloist) to 90.95% (RAV) (Figure 4F). In GC1, the wild varieties of the ERF‐II subfamily were significantly higher than the cultivated and landrace varieties (Figure 4D and Table S11). In GC2, the ERF‐II group of wild variety was significantly higher than the cultivated and landrace varieties, while the AP2‐II, AP2‐III, and RAV family of wild variety was significantly lower than the cultivated and landrace varieties (Figure 4E and Table S11). In GC3, the Soloist family for cultivated and landrace varieties was significantly higher than the wild varieties (Figure 4F and Table S11). These results further reveal the complex differences in codon usage and GC content among different group families and varieties.

### 3.5 Core and Dispensable Analysis of the SiAP2/ERF Superfamily

**FIGURE 5:**
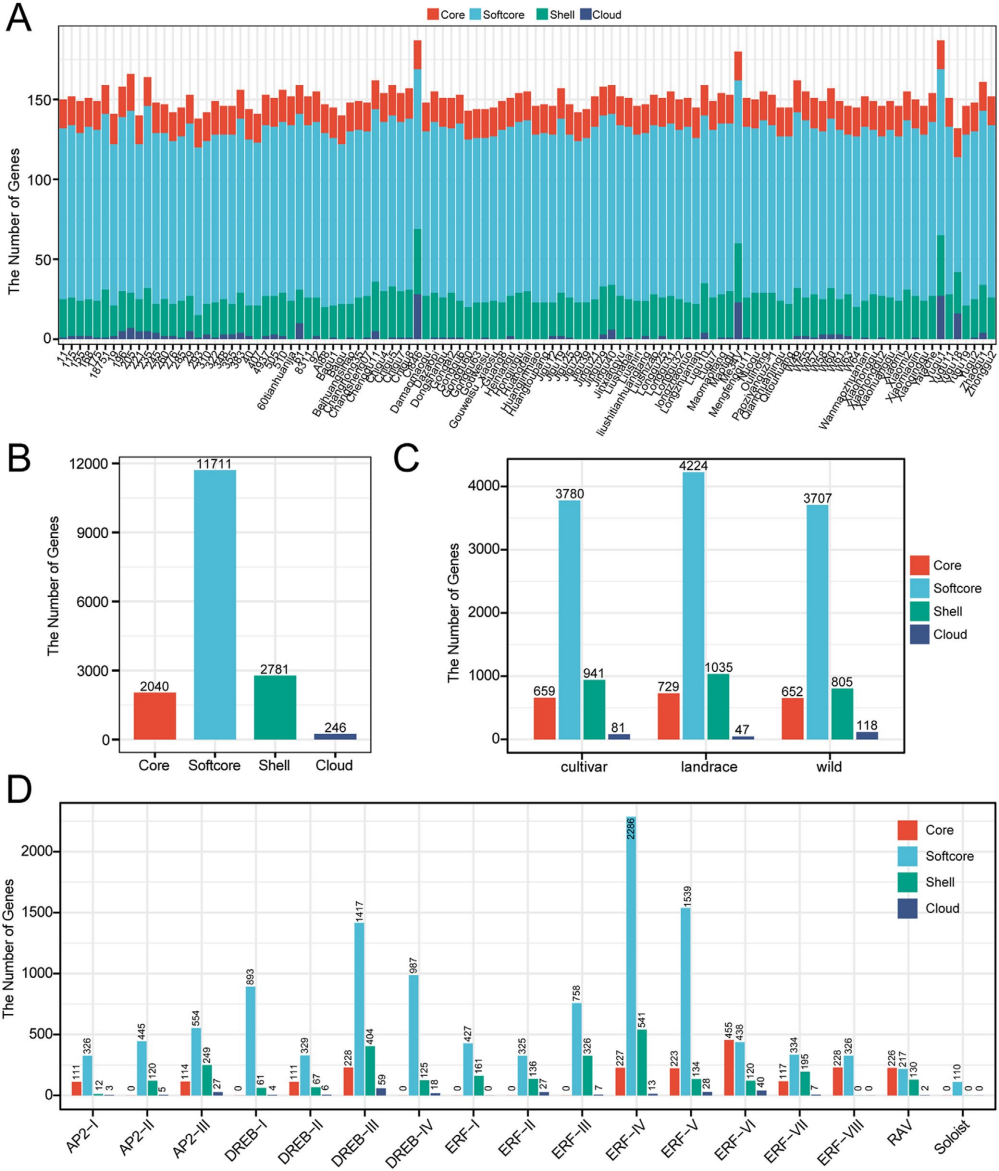
Pan‐genome analysis of the SiAP2/ERF superfamily. (A) Distribution of *AP2/ERF* genes in 111 foxtail millet accessions across five categories. (B) Total number of *SiAP2/ERF* genes in each category. (C) Distribution of *SiAP2/ERF* genes in each category across cultivars, landraces, and wild accessions. (D) Distribution of *SiAP2/ERF* genes in each category across different groups of SiAP2/ERF superfamily.

To explore the diversity within the SiAP2/ERF superfamily, we identified 225 OGGs using 111 *Setaria* accessions (Tables S8 and S9). The copy number analysis (Figure S5) indicated that OGG 142, OGG 143, OGG 144, and OGG 149 are absent in the wild population but are commonly present in both cultivated and landrace varieties. In contrast, the genes OGG 146, OGG 147, OGG 148, OGG 151, and OGG 154 are prevalent in the wild population yet are partially lost in cultivated and landrace varieties. Four distinct classes based on their presence across accessions, including 18 core, 107 softcore, and 100 other dispensable OGGs (51 shell and 49 cloud), were classified (Figure S6). The distribution of *SiAP2/ERF* genes across each category for each accession was shown in Figure 5A. It is clear that softcore genes are the most prevalent across all accessions, while cloud genes are the rarest (Figure 5A,B). The core and softcore consist of 125 OGGs, encompassing 13,751 genes, which account for 81.96% of the total (Figure 5B). This highlights the extensive conservation of the majority of *SiAP2/ERF* genes within the foxtail millet pan‐genome. The distribution of each gene type across different populations, including cultivars, landraces, and wild varieties, revealed that there are 4439 core‐like genes (core and softcore genes) in cultivars, 4953 in landraces, and 4359 in wild varieties, and these core‐like genes account for 81.29%, 82.07%, and 82.53% of the respective *SiAP2/ERF* gene pools in each population, respectively (Figure 5C), which suggests that the majority of *SiAP2/ERF* genes are conserved across diverse populations. Upon thorough examination of the gene counts and OGG distribution within each group of the SiAP2/ERF superfamily, it was observed that the ERF‐VI and RAV groups possess a greater number of core genes compared to other types (Figure 5D and Figure S6B), indicating a higher degree of conservation within these two groups. In contrast, the *AP2‐II*, *DREB‐I*, *DREB‐IV*, *ERF‐I*, *ERF‐II*, *ERF‐III*, and *Soloist* groups lack core genes, predominantly consisting of softcore genes.

### 3.6 Analyses of Natural Selection of SiAP2/ERF

**FIGURE 6:**
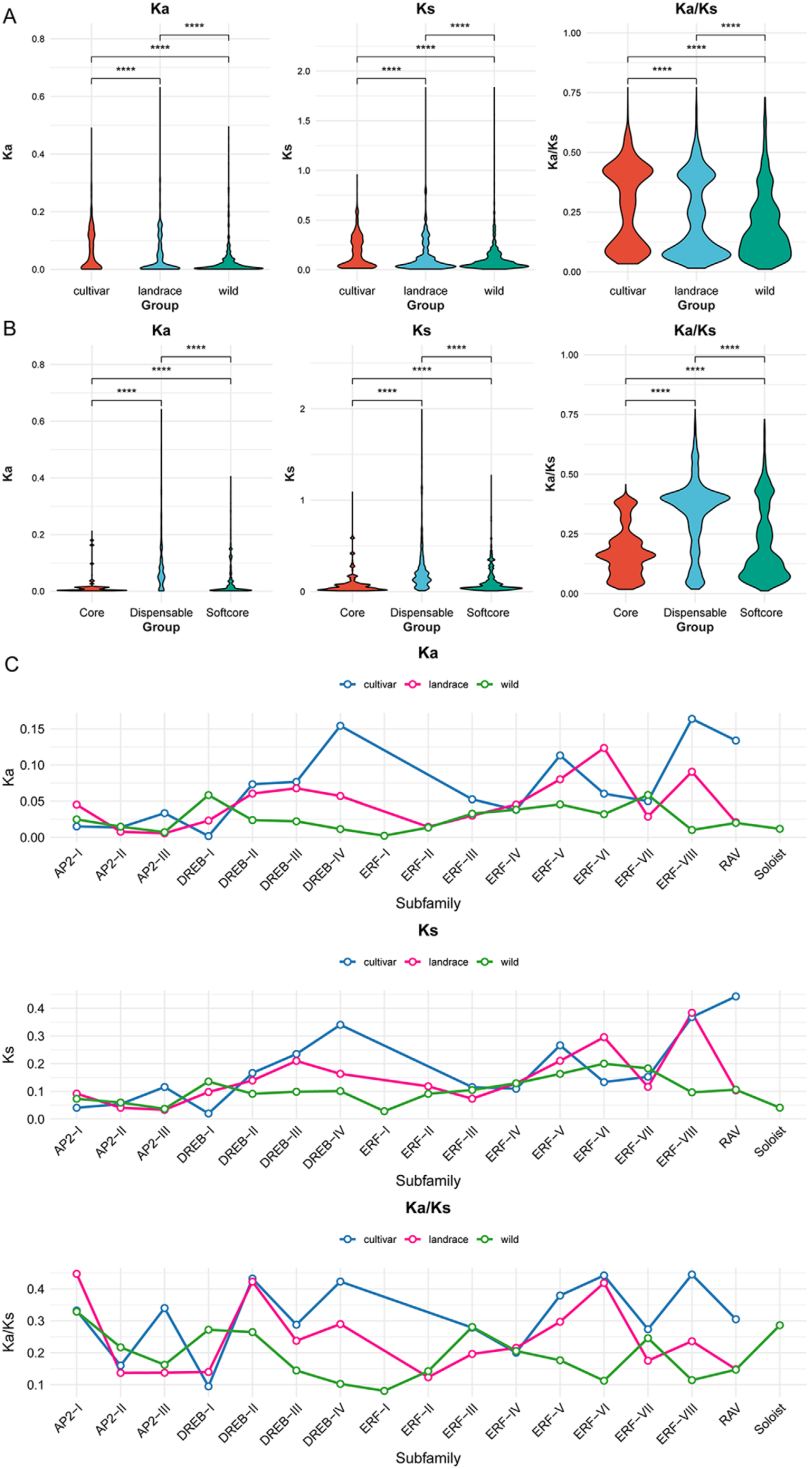
Analysis of natural selection on SiAP2/ERF superfamily at the pangenome level. (A) Comparison of Ka, Ks, and Ka/Ks for *SiAP2/ERF* genes across different populations. (B) Comparison of Ka, Ks, and Ka/Ks for AP2/ERF genes across core, dispensable, and softcore. (C) Variation in Ka, Ks, and Ka/Ks for different AP2/ERF groups across different populations. ****p < 0.0001.

The ratio of nonsynonymous (Ka) to synonymous (Ks) substitutions (*ω*) is a crucial metric for assessing selective pressures on genes, where *ω* < 1, *ω* = 1, and *ω* > 1 indicate purifying selection, neutral selection, and positive selection, respectively. To evaluate the impact of natural selection on the *AP2/ERF* gene family in foxtail millet, we calculated the Ka, Ks, and Ka/Ks values with *p* value < 0.01 for each OGG, and the results revealed that all *SiAP2/ERF* genes exhibit a Ka/Ks ratio less than 1 (Figure 6A). Significant variations in Ka, Ks, and Ka/Ks ratios were observed among the three populations, with the wild species showing a particularly lower Ka/Ks ratio of 0.195 compared to the landraces varieties at 0.225 and cultivated at 0.280 (Figure 6A). Despite there being genetic differences among various populations, the *SiAP2/ERF* genes were all under the influence of strong purifying selection.

Upon examining the selective pressures on core, softcore, and dispensable (shell, cloud) *SiAP2/ERFs*, we found that dispensable *SiAP2/ERF* genes have a notably higher average Ka of 0.084 and Ks of 0.231. In contrast, softcore *SiAP2/ERF* genes have Ka and Ks values of 0.034 and 0.111, respectively, while core *SiAP2/ERF* genes have values of 0.017 and 0.082 (Figure 6B). The Ka/Ks ratio for dispensable *SiAP2/ERFs* reached 0.334, which is significantly higher than those of the softcore (0.204) and core (0.173) (Figure 6B). The observations indicate that compared to the core and softcore *SiAP2/ERF* genes, the dispensable *SiAP2/ERF* genes typically experience relatively less selective pressure. The Ka/Ks ratio of the softcore genes is slightly higher than that of the core genes, further supporting that the core *SiAP2/ERF* genes are under more stringent purifying selection than noncore genes.

Upon evaluating the selective pressures across various AP2/ERF subfamilies, significant differences in Ka and Ks values were observed among different populations (Figure 6C). Specifically, within the DREB‐IV, ERF‐VIII, and RAV subfamilies, cultivars displayed elevated Ka values. In contrast, the ERF‐VI subfamily showed increased Ks values in landraces. A similar trend was observed in the DREB‐I subfamily, where wild species had higher Ks values. These findings underscore the diverse selective pressures influencing these gene subfamilies in distinct populations. When examining the Ka/Ks ratio across various SiAP2/ERF subfamilies, our analysis indicated that the majority of subfamilies in cultivars have higher Ka/Ks values than landraces and wild species, with notable variations among subfamilies from the lowest DREB‐I subfamily (0.095) to the highest ERF‐VIII subfamily (0.445). For landraces, the Ka/Ks ratio varied from 0.123 in the ERF‐II subfamily to a highest of 0.447 in the AP2‐I subfamily, and the ratio for AP2‐I was higher than that of cultivars and wild species. In wild species, the Ka/Ks ratio ranged from a minimum of 0.081 in the ERF‐I subfamily to a maximum of 0.329 in the AP2‐I subfamily, and the DREB‐I subfamily demonstrated the highest Ka/Ks ratios among the three populations. These results suggest that the selective pressures on different subfamilies vary among populations, highlighting the diverse evolutionary responses of these genes to environmental adaptations in different genetic backgrounds.

### 3.7 Evolution Analysis of *SiAP2/ERF* Gene Family

To study evolutionary mechanisms underlying *SiAP2/ERF* gene expansion, we categorized the *SiAP2/ERF* gene into four duplication types across 111 species and tallied the count of genes with various duplication types within each OGG (Table S8). The heatmap illustrating these findings is presented in Figure S7. Our analysis revealed that core and softcore genes are predominantly derived from WGD events, with a total of 60 OGGs, followed by the dispersed duplication type, which accounts for 44. For cloud and shell OGGs, 20.41% of the cloud OGGs and 15.69% of shell OGGs exhibit a greater prevalence of tandem duplications, which is higher than core (5.56%), softcore (7.48%) type.

**FIGURE 7:**
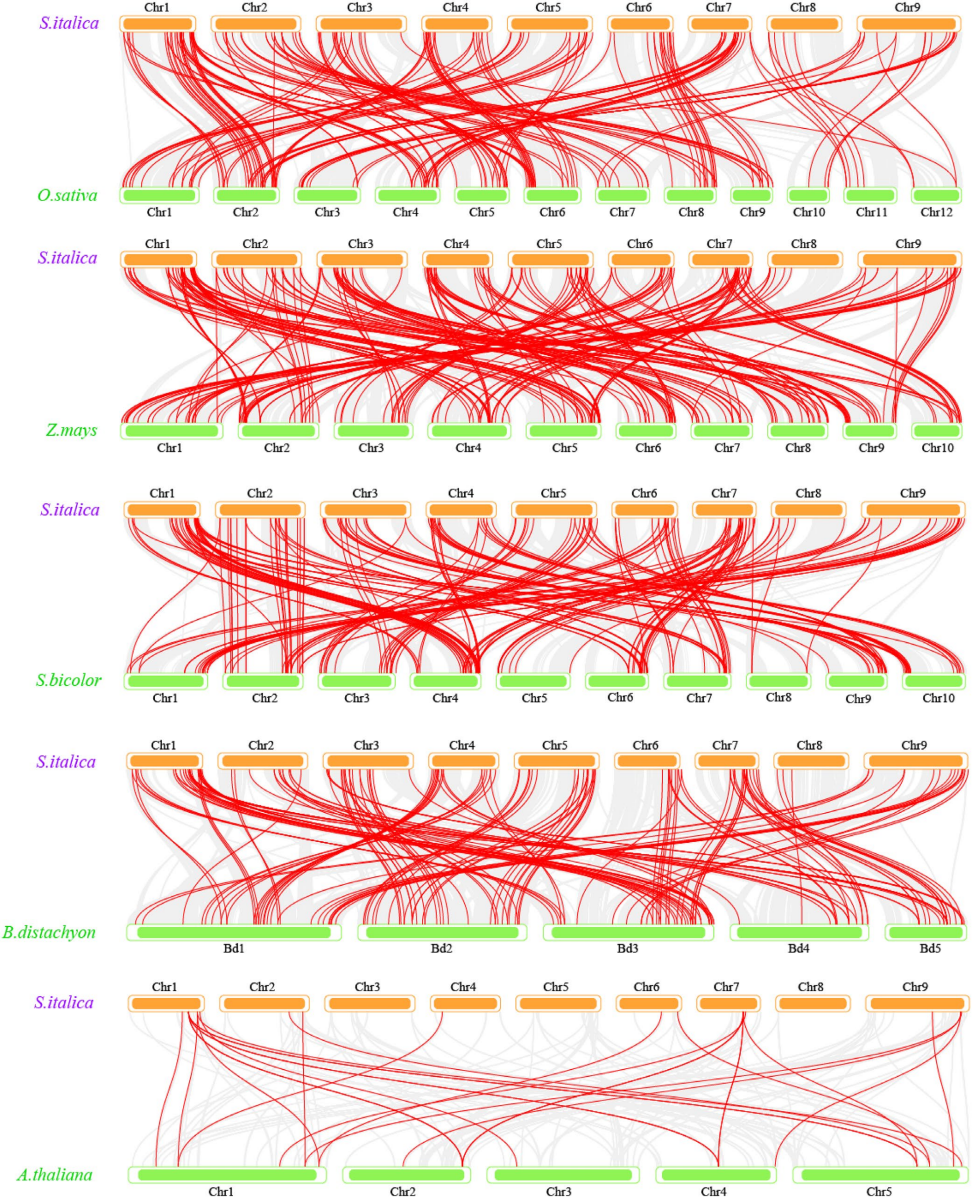
Collinearity analysis of *SiAP2/ERF* gene families between Yugu1 and other five plants. The syntenic *AP2/ERF* gene pairs were highlighted in red lines.

To gain insights into the evolutionary relationships of *SiAP2/ERF* genes across various plant species, we conducted a collinearity analysis between the foxtail millet (Yugu1) *SiAP2/ERF* genes and their homologs from other species, including rice, maize, sorghum, *Brachypodium distachyon* , and *A. thaliana* (Figure 7 and Table S12). Our findings revealed that the lowest number of collinear gene pairs was observed between foxtail millet and *A. thaliana* (26 pairs). In contrast, the highest number of gene pairs was found between foxtail millet and maize, totaling 246 pairs. Additionally, foxtail millet shared 157, 182, and 201 collinear gene pairs with *B. distachyon* , rice, and sorghum, respectively (Table S12). Among these collinear pairs, 107 foxtail millet *SiAP2/ERF* genes exhibited collinearity with all four other Poaceae species, comprising 17 AP2, 40 DREB, 46 RRF, 3 RAV, and 1 Soloist gene (Table S12). Moreover, of these, 15 *SiAP2/ERF* genes, including 4 DREB‐I (*SiDREB‐11*, *SiDREB‐34*, *SiDREB‐40*, *SiDREB‐56*), 5 DREB‐III (*SiDREB‐6*, *SiDREB‐15*, *SiDREB‐42*, *SiDREB‐45*, *SiDREB‐49*), 3 ERF‐IV (*SiERF‐11*, *SiERF‐12*, *SiERF‐74*), 1 ERF‐V (*SiERF‐9*), and 2 ERF‐VI (*SiERF‐14*, *SiERF‐93*), displayed collinearity across all species, suggesting the conservation of these genes across different species. The Ka/Ks analysis of these collinear genes indicated that values were less than 1 for all species, implying that the AP2/ERF gene family has been under strong purifying selection during species evolution (Table S12).

### 3.8 Expression Patterns of the SiAP2/ERF Gene Family in Response to Drought Memory

**FIGURE 8:**
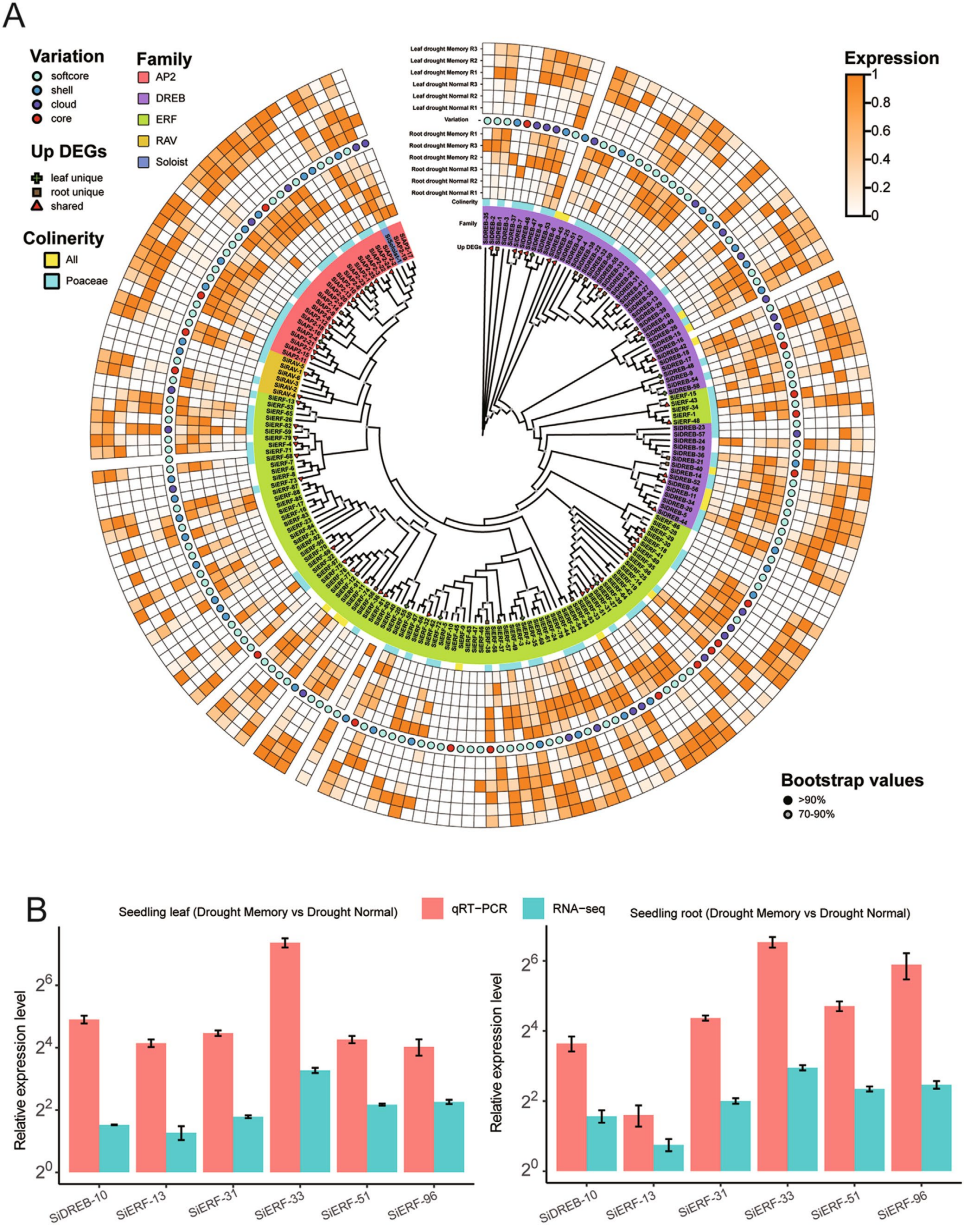
Expression patterns of *SiAP2/ERF* superfamily at second drought stress. (A) The heatmap shows the expression levels of *SiAP2/ERF* genes in Yugu1 leaf and tissues. (B) qRT‐PCR validation of differentially expressed genes identified by RNA‐seq. Gene expression levels measured by RNA‐seq are presented as log_2_ (fold change) (Drought Memory/Drought Normal), whereas qRT‐PCR results are expressed as relative expression levels calculated using the $2^{-\Delta \Delta C_{t}}$ method.

In leaves, the proportion of upregulated genes within the SiAP2/ERF family is 34.76%, whereas in roots, this proportion slightly increases to 37.43%. We investigated the expression patterns within each gene family (Figure 8A). We found that 17 of the 25 SiAP2 genes (68%) were significantly upregulated in leaves or roots during the second drought, highlighting the AP2 family's vital role in drought memory. In the DREB family, 27 genes were upregulated, accounting for 46.55% of the total and primarily located in the DREB‐III subfamily. Specifically, 14 genes were upregulated in both leaves and roots, while the remaining 13 genes showed tissue specificity, with 9 genes upregulated in roots and 4 genes specifically upregulated in leaves, all belonging to the DREB‐III subfamily. Additionally, two RAV genes (*SiRAV‐1* and *SiRAV‐4*) were significantly upregulated and expressed in both leaves and roots. In the ERF family, 34 genes were upregulated, representing 35.05% of the total. Notably, *SiERF‐51* and *SiERF‐96* stood out as the most highly expressed genes among all *SiAP2/ERF* genes, with significant upregulation in both roots and leaves and an upregulation fold change exceeding 18. These two genes are likely crucial for drought memory, with *SiERF‐51* also showing synteny with four other monocotyledonous plants.

Next, we investigated the enrichment of upregulated genes across different types of genomic variation (Figure S8). The results reveal that upregulated genes are significantly enriched among the core *SiAP2/ERF* genes. Specifically, in leaves, 8 core genes are upregulated, representing 44.44% of all core genes. In roots, the enrichment is even more pronounced, with 10 upregulated core genes accounting for 55.56% of the total. Notably, 7 core genes are upregulated in both leaves and roots, including *SiAP2‐7*, *SiAP2‐13*, *SiERF‐18*, *SiERF‐48*, *SiERF‐51*, *SiRAV‐1*, and *SiRAV‐4*. This finding suggests that these genes may play a crucial role in the drought memory response. In the softcore gene set, the proportion of upregulated genes is also higher than the overall distribution, with 42 upregulated genes in leaves (38.4%) and 44 in roots (40.38%). This indicates that upregulated genes are relatively lower enriched in the softcore gene set as well. For the cloud genes, the proportion of upregulated genes is 29.63% in leaves and 37.04% in roots, while in the shell genes, the proportions are 23.68% in leaves and 21.05% in roots, showing no enrichment in upregulated genes.

Furthermore, we examined the 107 *SiAP2/ERF* genes that exhibited synteny between foxtail millet and four other grass species, finding that 57 of these genes were upregulated in the drought memory group (second drought) in leaf or root tissues, representing 53.27% of the total. Among these genes, seven *SiAP2/ERF* genes were also syntenic with *A. thaliana* . Specifically, four genes (*SiDREB‐56*, *SiDREB‐42*, *SiDREB‐45*, and *SiERF‐11*) were upregulated and shared in both tissues, two genes were upregulated in roots (*SiDREB‐40* and *SiERF‐12*), and one gene (*SiDREB‐49*) was specifically upregulated in leaves. These genes are evolutionarily conserved and likely play a crucial role in responding to drought stress.

We validated the highly expressed core conserved gene *SiERF‐51*, which exhibited significant differential expression in both roots and leaves (Figure 8B). Additionally, we also confirmed the upregulation of other shared genes, such as *SiERF‐13*, *SiDREB‐10*, *SiERF‐33*, and *SiERF‐31*, verified by qRT‐PCR analysis, thereby ensuring the accuracy of our transcriptome data.

## 4 Discussion

Drought is one of the most significant abiotic stresses limiting crop growth and agricultural productivity (Qin et al. 2020; Terfa et al. 2025). Foxtail millet is highly drought‐tolerant and serves as a model species for drought resistance research (Qin et al. 2020; Terfa et al. 2025). Recent studies have demonstrated the existence of drought stress memory in plants (Yuan et al. 2024), with transcription factors, such as *AP2/ERF*, playing a crucial role in drought resistance memory (Agunbiade et al. 2024; Peer et al. 2025; Prusty et al. 2024; Terfa et al. 2025).

In this study, we employed ATAC‐seq technology to unravel the chromatin accessibility alterations in foxtail millet under normal and second drought stress. The findings revealed that second drought stress significantly modified the chromatin accessibility patterns in both the leaves and roots of foxtail millet, with a particularly notable increase in peak numbers in the drought stress group (Figure 1). This suggests that after experiencing the initial drought, plants undergo persistent epigenetic changes in their chromatin state, enabling a more rapid response in subsequent drought events. Such chromatin accessibility changes may provide more opportunities for transcription factor binding, thereby regulating the expression of drought‐responsive genes. Similar studies in *A. thaliana* and maize have also observed significant chromatin accessibility changes under abiotic stress (Han et al. 2025; Raxwal et al. 2020). Notably, AP2/ERF TFs were the most enriched transcription factors in both leaves and roots (Figure 1), not only supporting the significance of the AP2/ERF family in drought response but also implying that these specific transcription factors may play important roles in the formation of drought memory. This prompts us to focus on the characteristics, expression, and evolutionary patterns of the AP2/ERF superfamily in *foxtail millet*.

In the complete telomere‐to‐telomere genome of Yugu1, a total of 187 *AP2/ERF* transcription factor family members were identified, surpassing the 171 members identified in the draft genome previously reported (Lata et al. 2014). In addition, we identified one Soloist gene, which was not present in previous versions. This underscores the importance of utilizing complete genome assembly for accurate gene family identification. Transcriptomic analysis reveals that a substantial number of DEGs are responding to second drought stress in both leaves and root tissues (Figure 2), and the extensive shared gene expression patterns between leaves and roots may serve as the foundation for plants to coordinate their response to drought stress (Min et al. 2020). More specifically, 80 *SiAP2/ERF* genes were upregulated in second drought stress, and of which 55 were shared in both leaf and root tissues. This indicates that the AP2/ERF family holds a central position in regulating the expression of drought‐responsive genes. Numerous studies have elucidated the association between *AP2/ERF* genes and drought tolerance. For instance, *PtoERF15* in poplar (Kong et al. 2023), *GmERF3* in soybean (Zhang et al. 2009), *ZmDREB2A* in maize (Meena et al. 2022), and *TaERF87* in wheat (Du et al. 2023) have been identified as enhancers of drought resilience. These genes modulate hormone signaling, proline biosynthesis, and the expression of stress‐responsive genes, offering pivotal targets for enhancing crop resistance to abiotic stresses. Collectively, these insights consistently underscore the indispensable role of the AP2/ERF family in the plant's memory of drought stress.

The pan‐genome approach overcomes the limitations of a single reference genome by conducting whole‐genome sequencing of multiple samples, systematically characterizing the structural variations and copy number variations in cereal crops, and revealing their key associations with agricultural traits (Tao et al. 2019). This approach allows for a more accurate assessment of the dynamic changes within gene families and helps to overcome reference genome bias (Huang et al. 2025). The *AP2/ERF* gene family is categorized into various subfamilies based on the count and sequences of AP2 and B3 domains. Our classification approach integrates phylogenetic analysis with domain counts to define subfamily groupings. This method has produced a comprehensive and reliable catalog of 16,778 AP2/ERF genes across 111 foxtail millet accessions, organized into 17 groups (Figure 3). Comparing the distribution of these groups reveals that the ERF‐I, ERF‐II, and Soloist families are especially prevalent in both cultivated and landrace varieties. This suggests that they may have been selectively amplified during domestication and may have played an important role in enhancing agricultural traits. Expression analysis revealed more than 60% of the AP2 family is highly enriched with drought memory responsive genes. Codon usage bias refers to the nonrandom selection of synonymous codons in specific genes or species, and it can impact translation efficiency and accuracy, protein folding, and biological functions (Liu et al. 2021; C. H. Yu, Dang, et al. 2015). We analyzed the codon usage bias in *SiAP2/ERF* genes across various groups and populations and found that evolution and domestication have led to shifts in codon usage preferences (Figure 4). Soloist family's preference for UAG and UUG codons, in contrast to other families' use of CUG, AUC, CCG, GUG, CGC, GGC, and CUC, suggests a divergence in evolutionary strategies. The clustering of RSCU scores within gene families across populations indicates a conserved codon usage preference, potentially linked to similar expression demands or functional constraints. The variation in CAI values, particularly the Soloist family's lower values in wild populations, implies differences in gene expression optimization between domesticated and wild varieties. The ENC values, which measure codon usage bias, also show significant differences among groups, with some subfamilies exhibiting higher values, suggesting a potential impact on gene expression efficiency. These findings underscore the complex interplay between genetic variation, codon usage, and evolutionary adaptation in *SiAP2/ERF* genes, highlighting the need for further research to understand the functional implications of these biases in different environments and genetic backgrounds.

The exploration of diversity within the S*iAP2/ERF* superfamily using 111 Setaria accessions has identified 225 OGGs, categorized into core, softcore, shell, and cloud based on their prevalence across accessions (Figure 5). This stratification reveals a significant conservation of core‐like (core and softcore) genes, which constitute 81.96% of the total gene count, indicating a robust genetic foundation shared across foxtail millet populations. The distribution analysis across cultivars, landraces, and wild varieties further emphasizes the conservation of core‐like genes, which maintain a consistent presence in each population. This conservation suggests a fundamental role for these genes in the biology of *Setaria*, possibly linked to essential functions that are critical for survival and adaptation across diverse environments. We observed that drought memory responsive genes are enriched in the core‐like gene type, but not in the shell and cloud genes. This suggests that these core genes, such as *SiAP2‐7*, *SiAP2‐13*, *SiERF‐18*, *SiERF‐48*, *SiERF‐51*, *SiRAV‐1*, and *SiRAV‐4*, may play important roles in drought regulation and further investigation will be needed in the future study.

The assessment of natural selection pressures through Ka/Ks ratios across these OGGs provides insights into the evolutionary dynamics of the *SiAP2/ERF* genes. The observed Ka/Ks ratios, predominantly less than 1, suggest that purifying selection is a dominant force shaping these genes (Figure 6). However, the variation in Ka/Ks ratios among different populations and gene types (core, softcore, dispensable) indicates that the intensity of selection varies. Dispensable genes exhibit a higher Ka/Ks ratio, suggesting that they are under less selective pressure compared to core and softcore genes, which aligns with research on the *bHLH* gene family in barley (Tong et al. 2025). The Ka/Ks ratios in cultivated varieties were higher for most subfamilies compared to landraces and wild species, suggesting that purifying selection may be relaxed or positive selection may be enhanced in cultivated varieties. This phenomenon is likely due to the selection for certain traits during domestication and breeding processes (Tao et al. 2022), leading to a higher frequency of fixation of nonsynonymous mutations in the populations of these varieties.

Collinearity analysis is crucial for studying gene family evolution and conservation (Tang et al. 2008). Through collinearity analysis of foxtail millet with rice, maize, sorghum, *B. distachyon* , and *A. thaliana* , it reveals the conservation and variation of *SiAP2/ERF* genes during evolution (Figure 7). The analysis revealed the lowest number of collinear gene pairs between foxtail millet and *A. thaliana* , with only 26 pairs, whereas the highest number was observed with maize, totaling 246 pairs. Among these comparisons, 107 *SiAP2/ERF* genes demonstrated synteny with four other Poaceae species, suggesting that these genes are highly conserved and may play a significant role in species. Transcriptional analysis revealed one core gene, *SiERF‐51*, that demonstrated the highest levels of expression and was significantly upregulated in both leaves and roots (Figure 8), which may be associated with drought memory responses. Future research should focus on the functional validation of these transcription factors to better understand their specific molecular mechanisms in drought memory and adaptation.

## 5 Conclusion

In this study, we integrated ATAC‐seq, RNA‐seq, and pan‐genome analysis to explore the response mechanisms of the *foxtail millet AP2/ERF* superfamily under repeated drought stress. A significant increase in chromatin accessibility peaks was observed in the leaves and roots of *foxtail millet* under secondary drought stress. The *AP2/ERF* were identified as the most enriched transcription factors in the differential chromatin regions. Transcriptome analysis further revealed that 80 *SiAP2/ERF* genes were upregulated in response to drought stress. Pan‐genome analysis unveiled the gene distribution characteristics of the foxtail millet *AP2/ERF* superfamily and their potential roles in adapting to various environmental stresses. Noncore genes exhibited higher Ka/Ks ratios, suggesting they may have experienced less selective pressure and possess greater evolutionary flexibility. Collinearity analysis revealed that a substantial number of foxtail millet *AP2/ERF* genes exhibited collinearity across all four other Poaceae species, indicating a high degree of conservation of these genes among different species. Natural selection indicated that all *SiAP2/ERF* genes are subject to purifying selection, which helps maintain the functional stability of the gene family. We identified core gene *SiERF‐51* as the most prominently upregulated gene during the second drought stress, underscoring its pivotal role in drought response pathways. This highlights the role of pan‐genome analysis in identifying key genes. In summary, this study not only reveals the pivotal role of the *AP2/ERF* family in drought repeat stress but also provides a wealth of genetic resources for further investigations into the molecular mechanisms of drought adaptation in foxtail millet, guiding the development of more resilient and productive varieties in the face of a changing climate.

## Author Contributions

T.L. conceived the study and designed the experiments. Experimental work was performed by T.L., W.L., Z.W., and J.L. Data analysis was conducted by T.L., H.W., X.Z., Y.C., J.Z., F.F., Y.W., and Y.S. The manuscript was drafted by T.L., W.L., and Y.S., with critical revisions by W.W. and J.H. All authors contributed to result interpretation, manuscript discussion, and approved the final version.

## Consent

The authors have nothing to report.

## Conflicts of Interest

The authors declare no conflicts of interest.

## Supporting information


**Figure S1:** Experimental design for drought stress treatments.
**Figure S2:** GO and KEGG enrichment of shared upregulated genes.
**Figure S3:** SiAP2/ERF superfamily classification based on *Arabidopsis thaliana* AP2/ERF.
**Figure S4:** Relative synonymous codon usage (RSCU) cluster analysis of different groups of SiAP2/ERF superfamily in different populations.
**Figure S5:** Copy number distribution for each OGG among three populations.
**Figure S6:** Group classification of all *SiAP2/ERF* genes in 111 accessions. (A) Distribution of OGGs in each category of pan gene family types. (B) Distribution of OGGs in each category across different groups of SiAP2/ERF superfamily.
**Figure S7:** The duplicate type distribution for each OGG.
**Figure S8:** DEG type distribution among variation type.

**Table S1:** Primer sequences used for qRT‐PCR validation of candidate genes.
**Table S2:** ATAC‐seq and RNA‐seq sequencing statistics.
**Table S3:** Motif enrichment in leaf tissue.
**Table S4:** Motif enrichment in root tissue.
**Table S5:** DEGs between Leaf drought normal and Leaf drought memory.
**Table S6:** DEGs between Root drought normal and root drought memory.
**Table S7:** Statistics of AP2/ERF superfamily in Yugu1 foxtail millet.
**Table S8:** Pangenome‐wide identification of AP2/ERF superfamily in foxtail millet.
**Table S9:** Cluster analysis all SiAP2/ERF genes in foxtail millet.
**Table S10:** Codon parameters for each of SiAP2/ERF genes.
**Table S11:** Values of codon parameters and results of significance tests across different populations.
**Table S12:** Ka/Ks values for each OGGs.
**Table S13:** Collinear gene pairs and Ka Ks results.
**Table S14:** SiAP2/ERF from Yugu1 gene expressions.

## Data Availability

The data that support the findings of this study are openly available in China National Center for Bioinformation database at https://ngdc.cncb.ac.cn/gsub/submit/bioproject/subPRO059645/overview, reference number PRJCA040542.
