# Supplementary material for: Integrating ATAC‐Seq and Pan‐Genomics Identifies Stress‐Memory AP2/ERF Hubs in Foxtail Millet
Source: Food Sci Nutr. 2025 Nov 29;13(12):e71109. doi: 10.1002/fsn3.71109 (PMC12663697; doi:10.1002/fsn3.71109)
Supplement: Supplementary file 1 — Figure S1: Experimental design for drought stress treatments. Figure S2: GO and KEGG enrichment of shared upregulated genes. Figure S3: SiAP2/ERF superfamily classification based on Arabidopsis thaliana AP2/ERF. Figure S4: Relative synonymous codon usage (RSCU) cluster analysis of different groups of SiAP2/ERF superfamily in different populations. Figure S5: Copy number distribution for each OGG among three populations. Figure S6: Group classification of all SiAP2/ERF genes in 111 accessions. (A) Distribution of OGGs in each category of pan gene family types. (B) Distribution of OGGs in each category across different groups of SiAP2/ERF superfamily. Figure S7: The duplicate type distribution for each OGG. Figure S8: DEG type distribution among variation type. [file FSN3-13-e71109-s001.docx]

**Supplementary Figures**


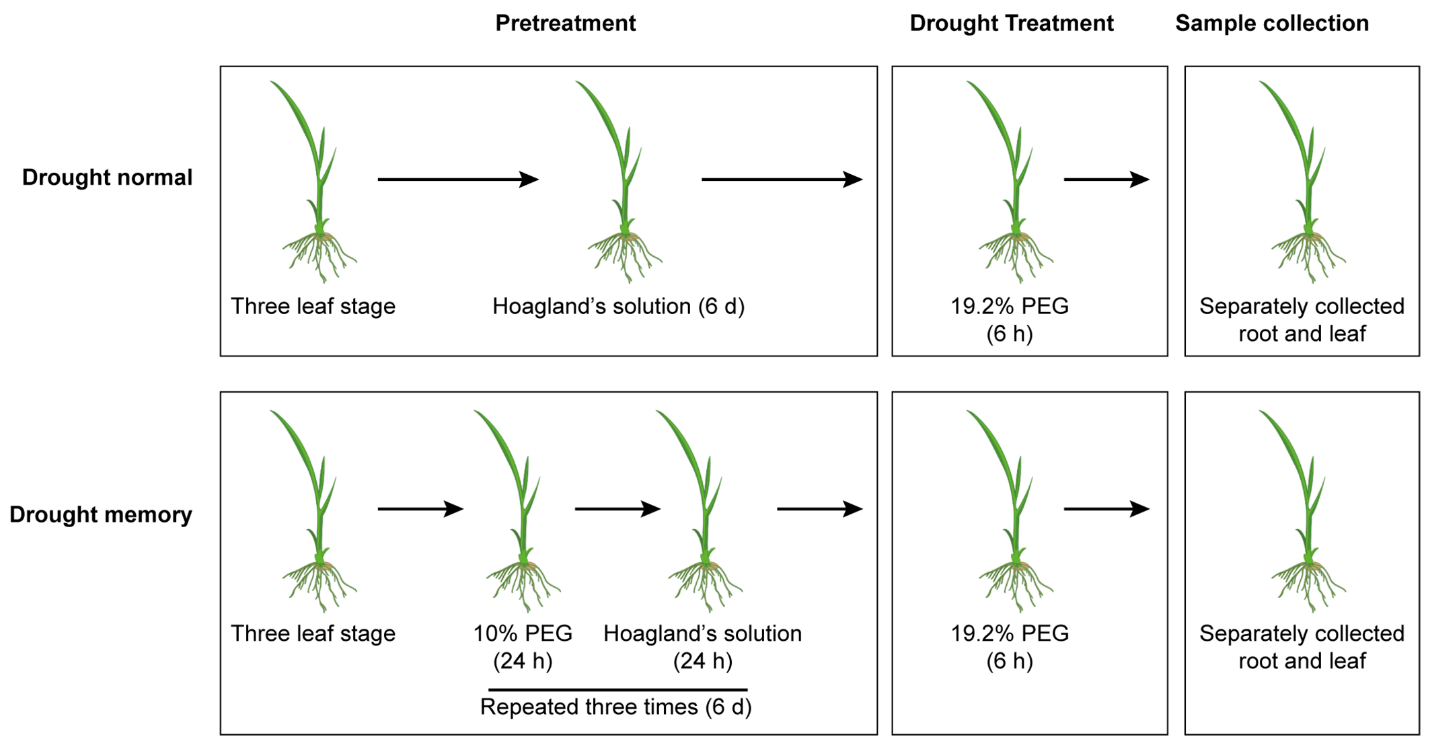


**Figure S1. Experimental design for drought stress treatments.**


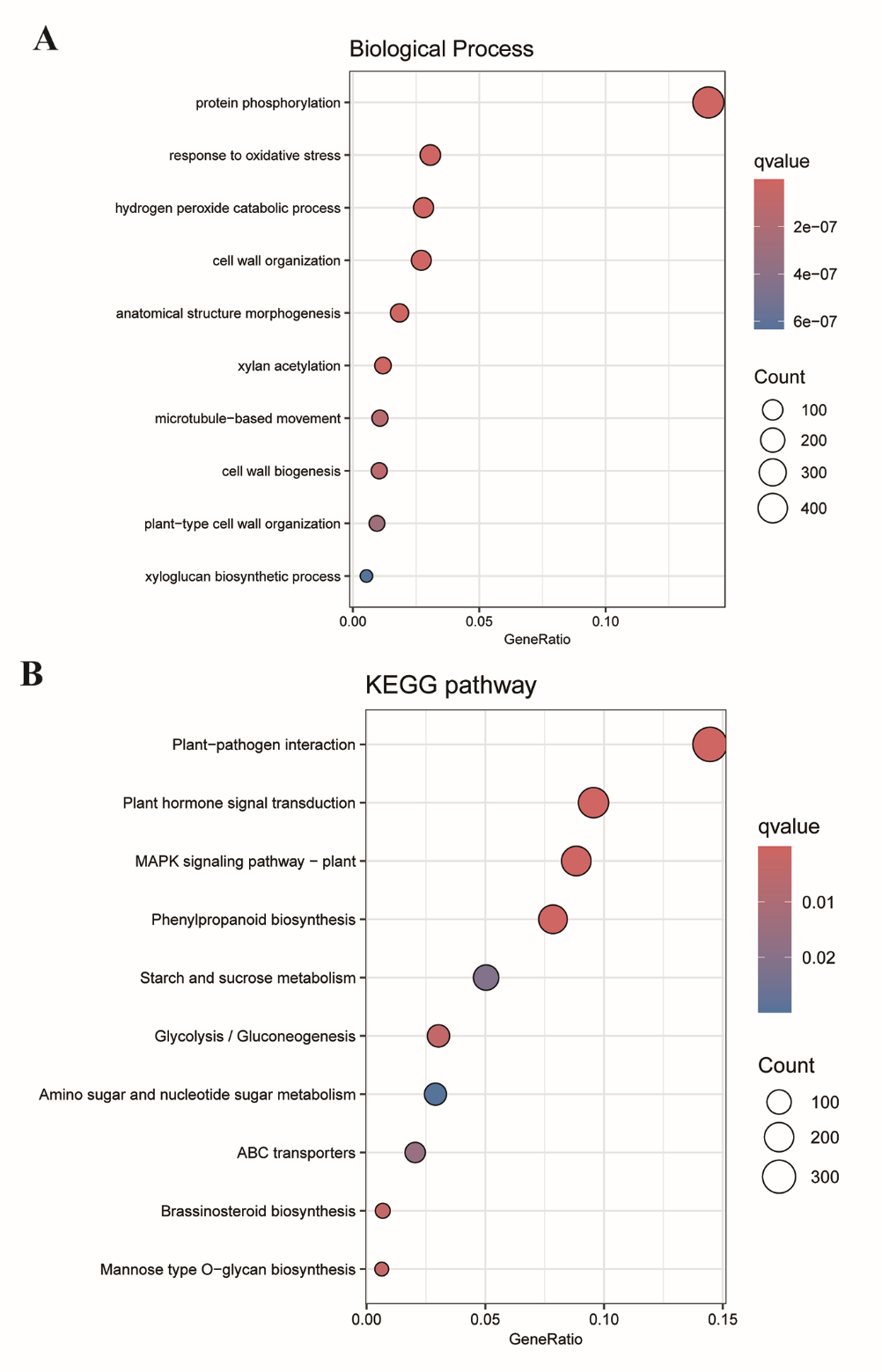


**Figure S2. GO and KEGG enrichment of shared upregulated genes**


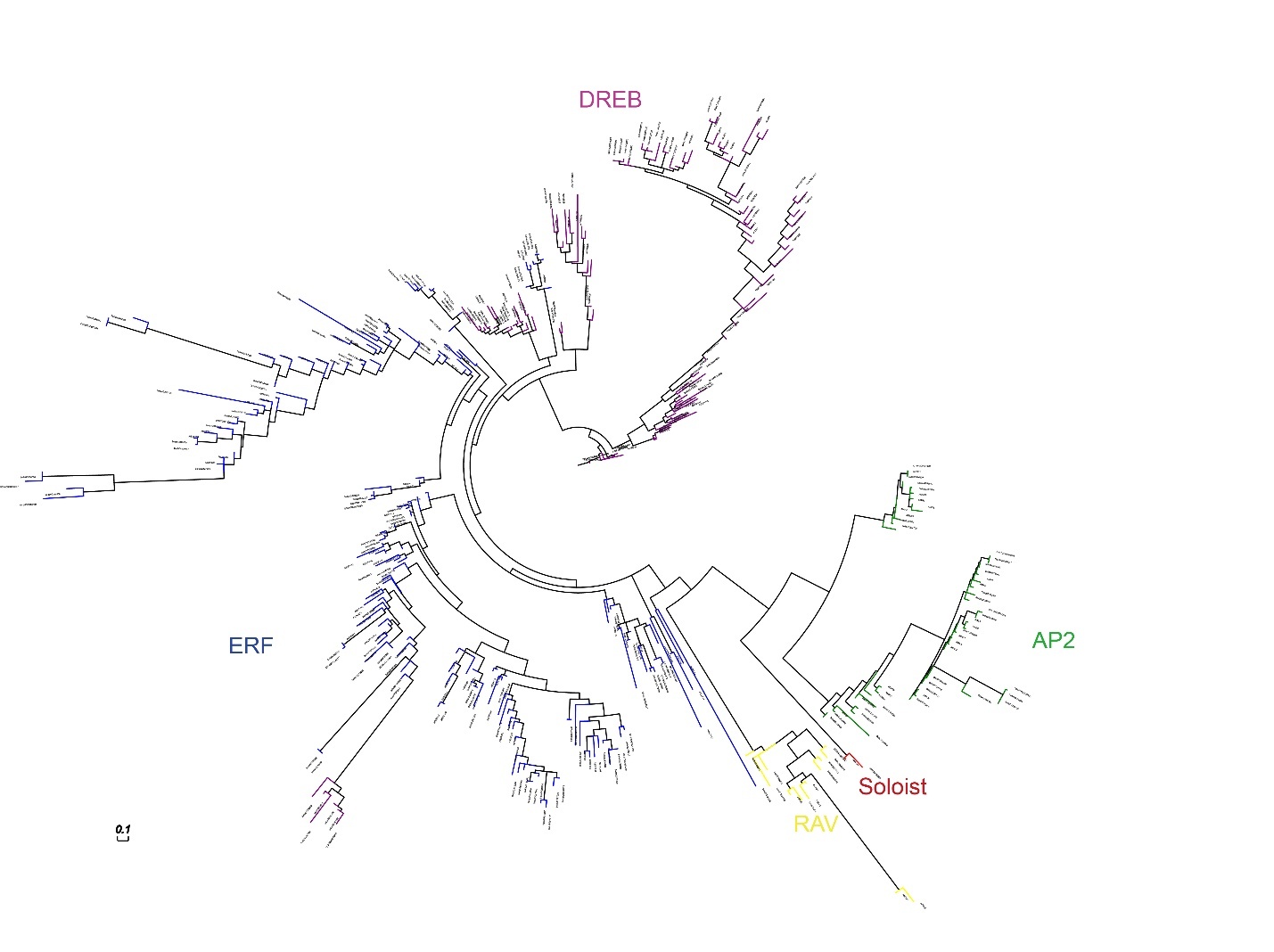
**Figure S3. SiAP2/ERF superfamily classification based on *Arabidopsis thaliana* AP2/ERF.**

**
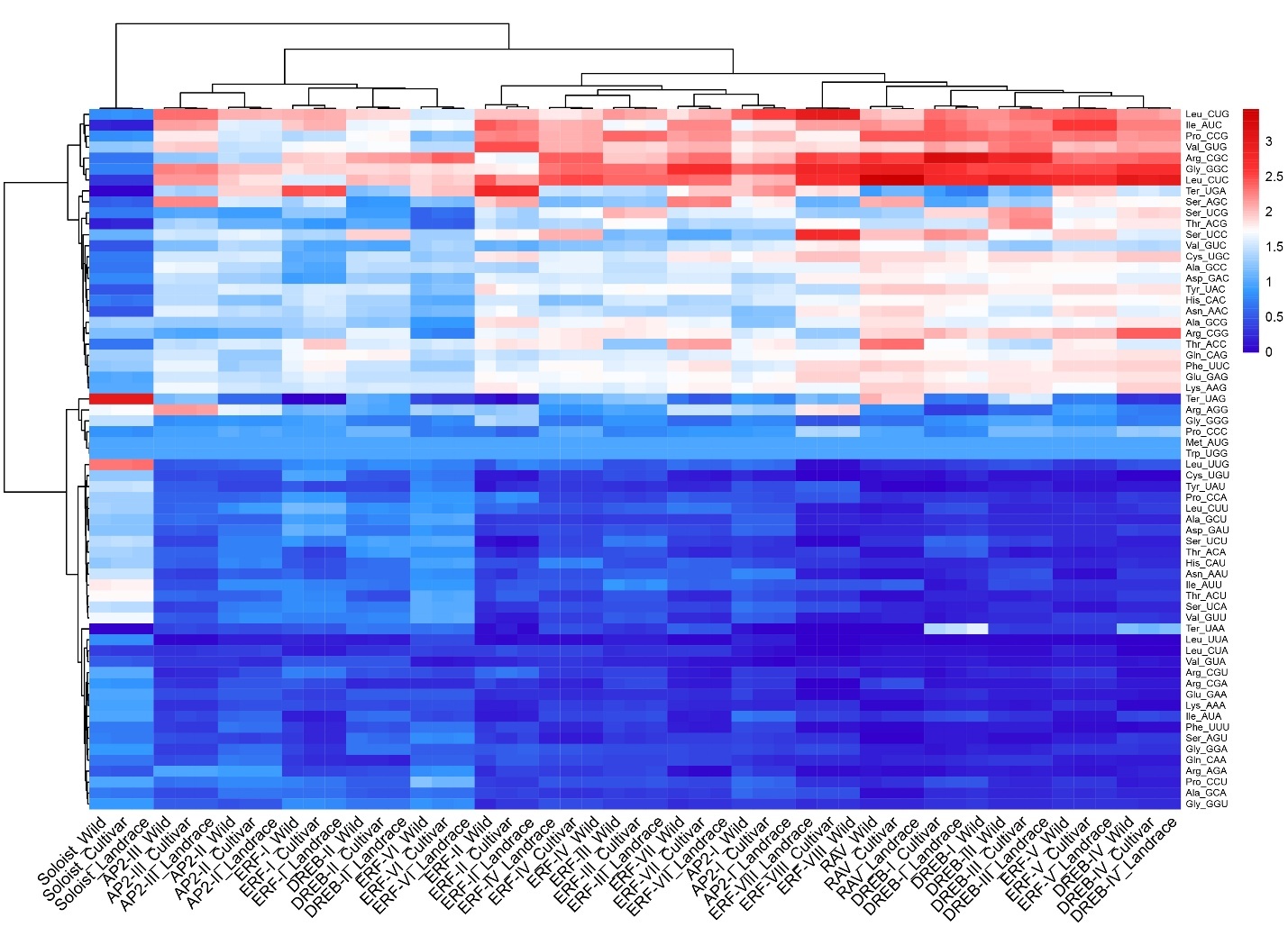
**

**Figure S4. Relative synonymous codon usage (RSCU) cluster analysis different groups of SiAP2/ERF superfamily in different populations.**


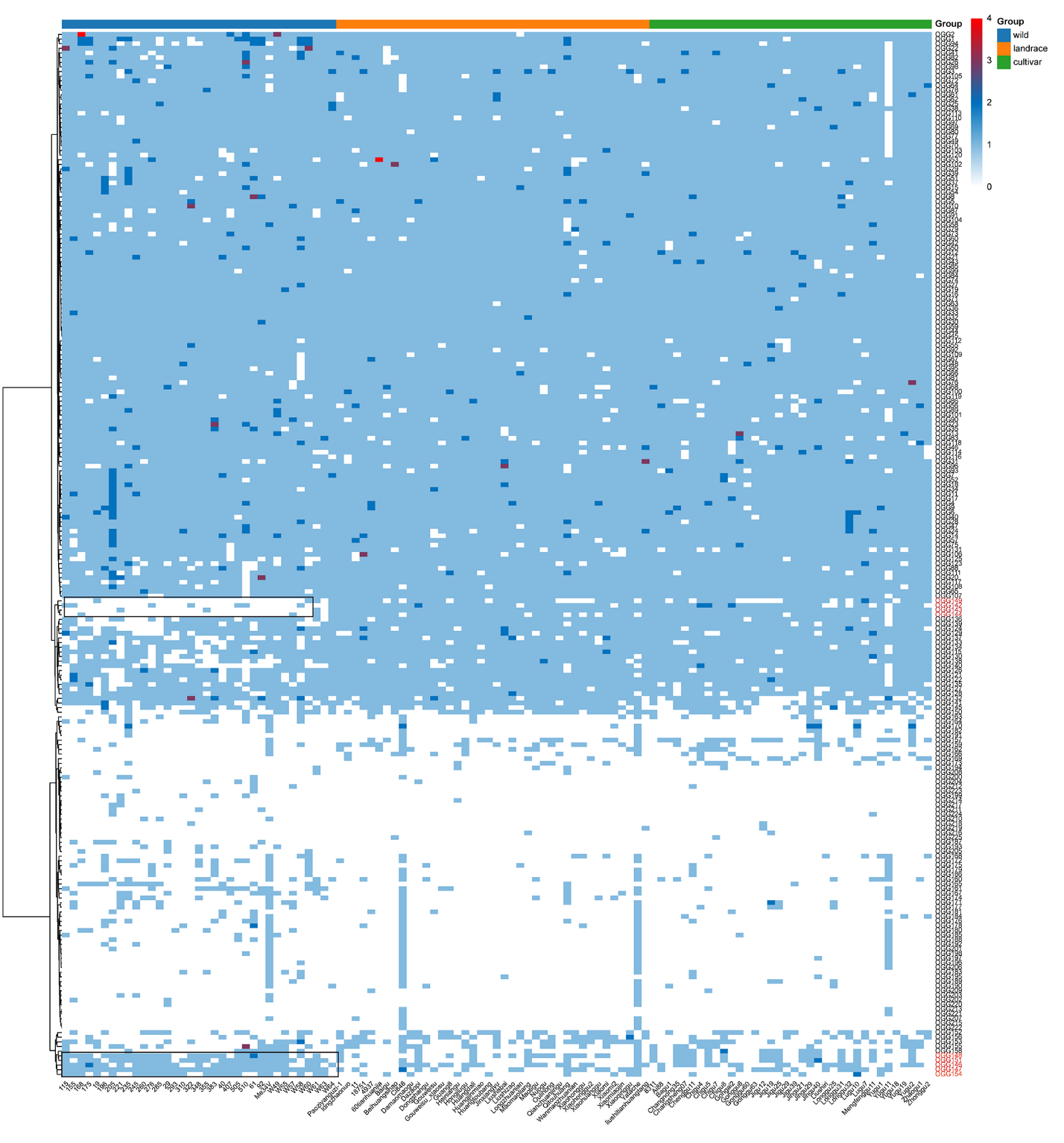


**Figure S5. Copy number distribution for each OGG among three populations**

**
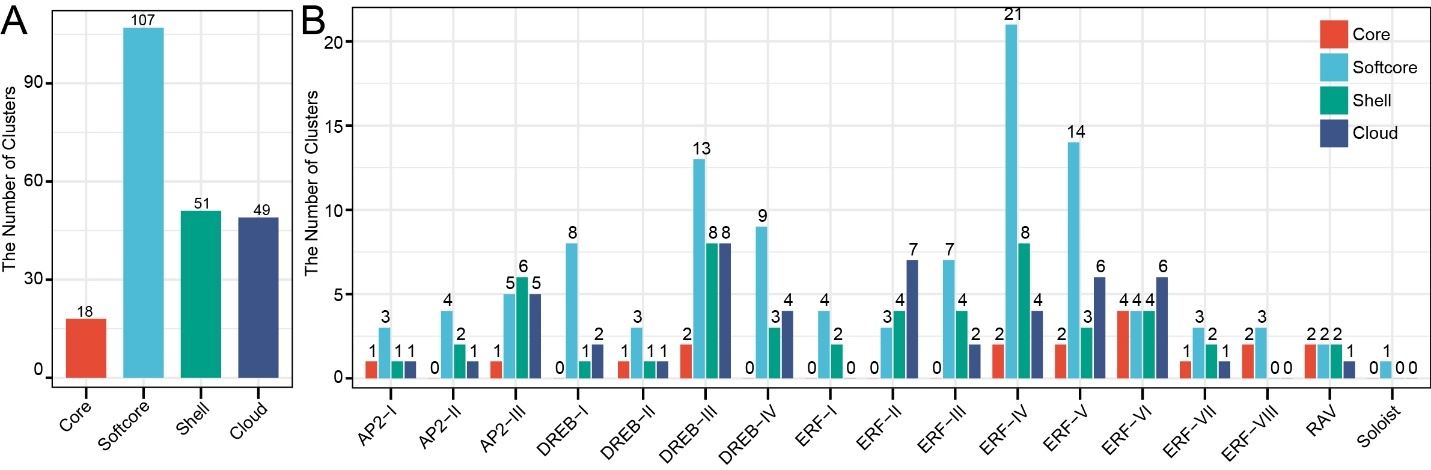
**

**Figure S6. Group classification of all *SiAP2/ERF* genes in 111 accessions.** (A) Distribution of OGGs in each category of pan gene family types. (B) Distribution of OGGs in each category across different groups of SiAP2/ERF superfamily.


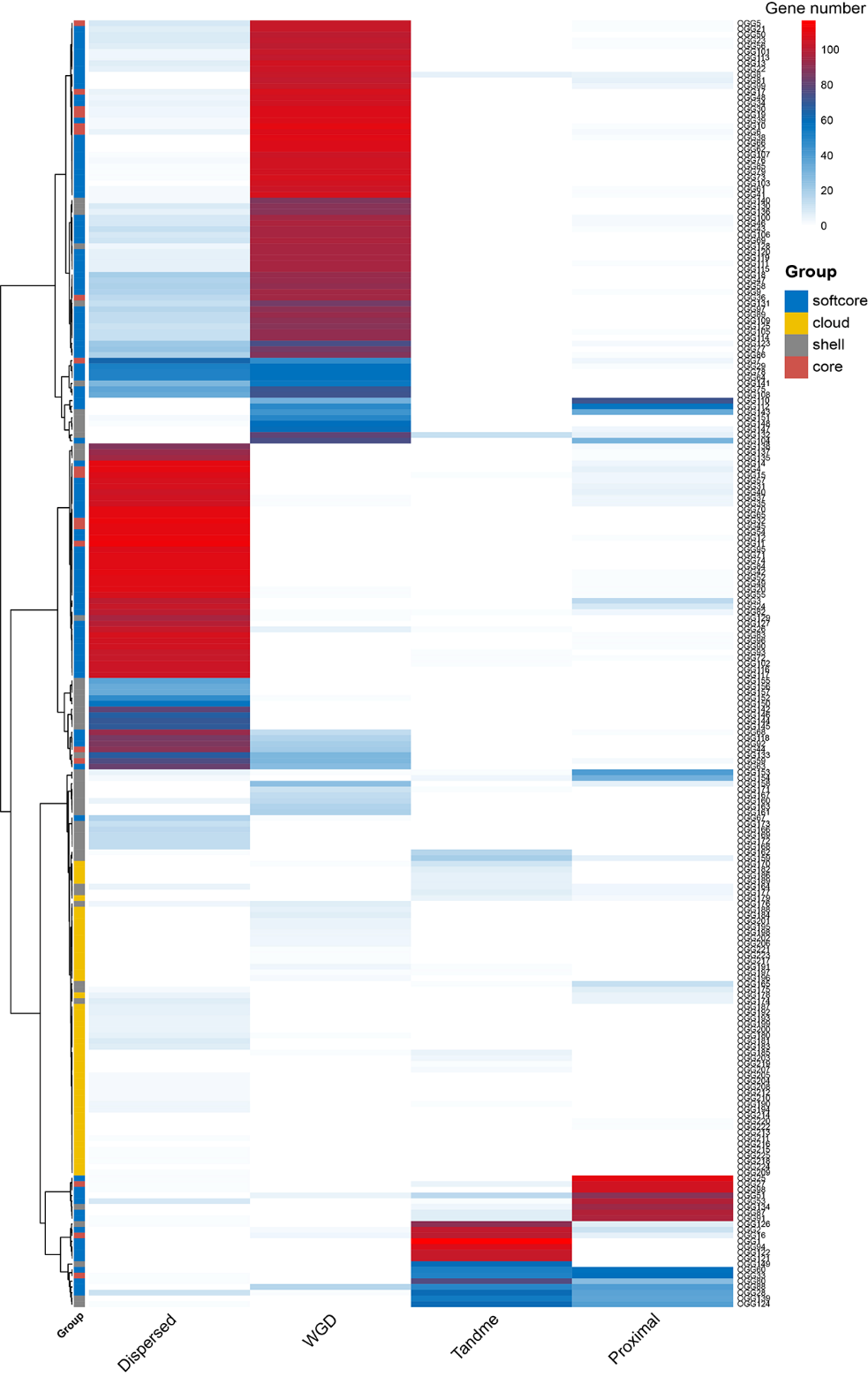


**Figure S7. The duplicate type distribution for each OGG**

*
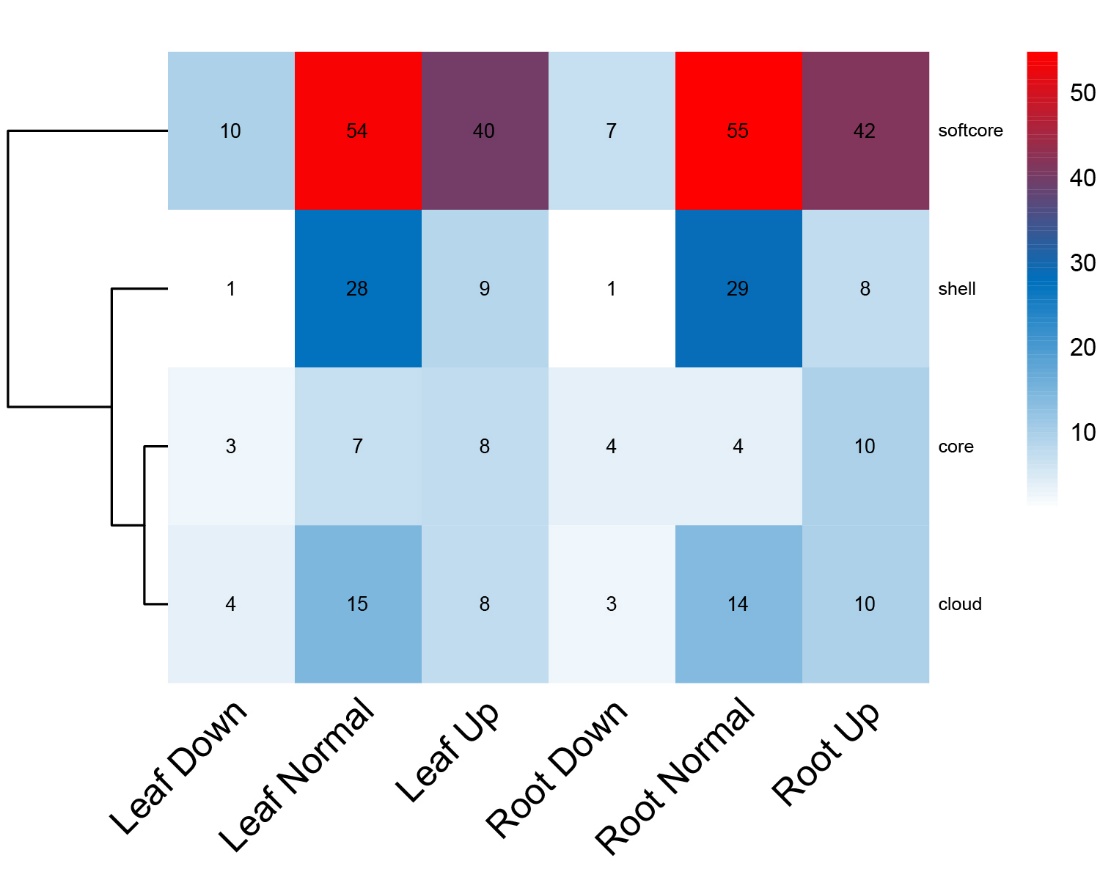
*

**Figure S8. DEG type distribution among variation type**
